# Energy metabolism in ALS: an underappreciated opportunity?

**DOI:** 10.1007/s00401-018-1835-x

**Published:** 2018-03-16

**Authors:** Tijs Vandoorne, Katrien De Bock, Ludo Van Den Bosch

**Affiliations:** 1 Department of Neurosciences, Experimental Neurology, KU Leuven-University of Leuven, Campus Gasthuisberg O&N 4, Herestraat 49, PB 602, 3000 Leuven, Belgium; 2 Laboratory of Neurobiology, Center for Brain & Disease Research, VIB, 3000 Leuven, Belgium; 3 Laboratory of Exercise and Health, Department of Health Sciences and Technology, ETH Zurich, Zurich, Switzerland

**Keywords:** Amyotrophic lateral sclerosis, Energy metabolism, Neuron-glia metabolic coupling, Mitochondria, Metabolic dysfunction, Metabolic treatment

## Abstract

Amyotrophic lateral sclerosis (ALS) is a relentlessly progressive and fatal neurodegenerative disorder that primarily affects motor neurons. Despite our increased understanding of the genetic factors contributing to ALS, no effective treatment is available. A growing body of evidence shows disturbances in energy metabolism in ALS. Moreover, the remarkable vulnerability of motor neurons to ATP depletion has become increasingly clear. Here, we review metabolic alterations present in ALS patients and models, discuss the selective vulnerability of motor neurons to energetic stress, and provide an overview of tested and emerging metabolic approaches to treat ALS. We believe that a further understanding of the metabolic biology of ALS can lead to the identification of novel therapeutic targets.

## Introduction

Amyotrophic lateral sclerosis (ALS) is a fatal neurodegenerative disorder characterized by the selective and progressive degeneration of motor neurons in the brain and spinal cord. Motor neuron deterioration leads to muscle weakness and results in death due to respiratory failure typically within 3–5 years after diagnosis [25]. In the Western world, the lifetime risk of developing ALS is estimated to be 1 in 400 [89].

ALS is a highly heterogeneous disease [187]; 5–10% of patients have a familial form in which inheritance almost exclusively occurs via an autosomal dominant Mendelian pattern. While over 120 potential ALS genes (http://alsod.iop.kcl.ac.uk/) have been identified, more than half of familial ALS patients carry mutations in either ‘*superoxide dismutase 1*’ (SOD1), ‘*TAR DNA binding protein*’ (TARDBP), ‘*fused in sarcoma*’ (FUS), or carry a hexanucleotide repeat expansion in an intronic region of the ‘*chromosome 9 open reading frame 72*’ (C9ORF72) gene [190]. Despite the genetic heterogeneity, most patients show cytoplasmic inclusions in motor neurons which stain positive for TDP-43, the protein product of *TARDBP* [136]. This suggests that similar pathogenic mechanisms may be present in different ALS subtypes. Although most ALS patients have no family history, unraveling the genetic basis of the disease led to an array of ALS models, put forth different processes believed to be involved in ALS pathogenesis, and led to various clinical trials [190]. Despite these efforts, translation of preclinical findings into effective therapeutic strategies remained poor. Riluzole and edaravone are the only FDA-approved drugs to treat ALS. Riluzole prolongs life by only a few months [12] and edaravone improves patient functionality scores in a subset of patients [165, 211]. Due to the unavailability of effective drugs, there is an urgent need for new treatment modalities in ALS.

A growing body of evidence shows dysregulated energy metabolism in ALS patients and models. Several of the metabolic abnormalities in ALS correlate to disease susceptibility and progression. Moreover, the remarkable vulnerability of motor neurons to energy depletion has become increasingly clear. In this review, we focus on how energy metabolism is impaired in ALS, and how motor neuron physiology contributes to their particular vulnerability to metabolic stress. We also discuss tested and emerging metabolism-centric therapeutic avenues for ALS.

## Systemic metabolism correlates to ALS disease course

Control of whole-body energy homeostasis, the balance between energy uptake and expenditure, is crucial to maintain stable body weight and hence overall health [105]. In ALS patients, energy homeostasis is imbalanced [57]. While energy uptake is often lowered [1], energy expenditure is suggested to be increased in a significant proportion of patients with ALS [21]. While this observation stems from predictive equations which still need validation in ALS patients and should, therefore, be interpreted with care [176], energy expenditure exceeds uptake in most ALS patients, leading to reduced fat depots [81]. Imbalanced energy homeostasis is also a consistent finding in different SOD1 [56, 62] and TDP-43 mouse models [30, 36]. Recently, the melanocortin pathway, a critical regulator of energy homeostasis and food intake in the hypothalamus [184], was hypothesized to contribute to imbalanced energy homeostasis in ALS patients [67] and mice [201]. However, reducing energy expenditure and inducing hyperphagia by targeting this pathway in mutant SOD1^G93A^ mice did not improve motor function or lifespan [53]. While the cause and importance of dysregulated energy homeostasis in human ALS remains to be established, body weight loss is an important prognostic factor in patients [149]. A lower pre-symptomatic body mass index has been reported in ALS patients [86, 126, 149] and the ALS risk is reduced up to 40% among obese individuals [138]. In agreement, increased prediagnostic body fat [65], subcutaneous fat [111], and serum leptin [135] were associated with a decreased risk of ALS mortality.

The majority of ALS patients suffer from hypolipidemia [215]. Of note, hypolipidemia is also present in mutant SOD1 mice [62, 98] and precedes clinical onset in mutant SOD1^G93A^ mice [98]. Whether hypolipidemia is also a preclinical feature in human ALS patients is difficult to assess, since diagnostic certainty is only reached in a progressed stage of the disease. In addition, elevated serum cholesterol and apolipoprotein E levels prolong survival and delay disease progression in ALS patients in most [52, 54, 103], but not all [31], studies, while statin treatment was associated with worsened outcome [224]. An additional study showed a positive correlation between blood lipids and respiratory function in ALS patients, potentially due to the decrease in CO_2_ production, which lowers the load on ventilatory muscles [27, 31].

Interestingly, ALS patients suffering from diabetes show a delay in the onset of motor symptoms for up to 4 years [87]. A large case–control study reported an estimated odds ratio for ALS association with diabetes of 0.61 (95% confidence interval: 0.46–0.80) [99]. Remarkably, type II diabetes was associated with a decreased risk of ALS (odds ratio 0.79, 95% confidence interval: 0.68–0.91) [127], while type I diabetes was associated with an increased risk (odds ratio 5.38, 95% confidence interval: 1.87–15.51) [194]. These data suggest that a potential protective effect is restricted to type II diabetes. Large longitudinal studies are required to determine whether insulin resistance (a hallmark of type II diabetes) per se has a protective effect against ALS or whether the protective effect is secondary to environmental and/or genetic factors that contribute to the development of type II diabetes. Moreover, ALS patients often develop insulin resistance during the course of the disease [154]. Since muscle tissue represents the major site of glucose consumption and storage, the development of insulin resistance during ALS is considered a consequence of muscle atrophy, although molecular evidence is still lacking. Even more, it has been suggested that deregulation of carbohydrate metabolism might contribute to ALS pathogenesis (see below).

Altogether, systemic metabolic defects in ALS correlate with disease duration and/or progression [21, 26, 81]. However, it remains to be determined whether and how these defects are causally connected to ALS pathogenesis.

## Motor neuron metabolism in health

**Fig. 1:**
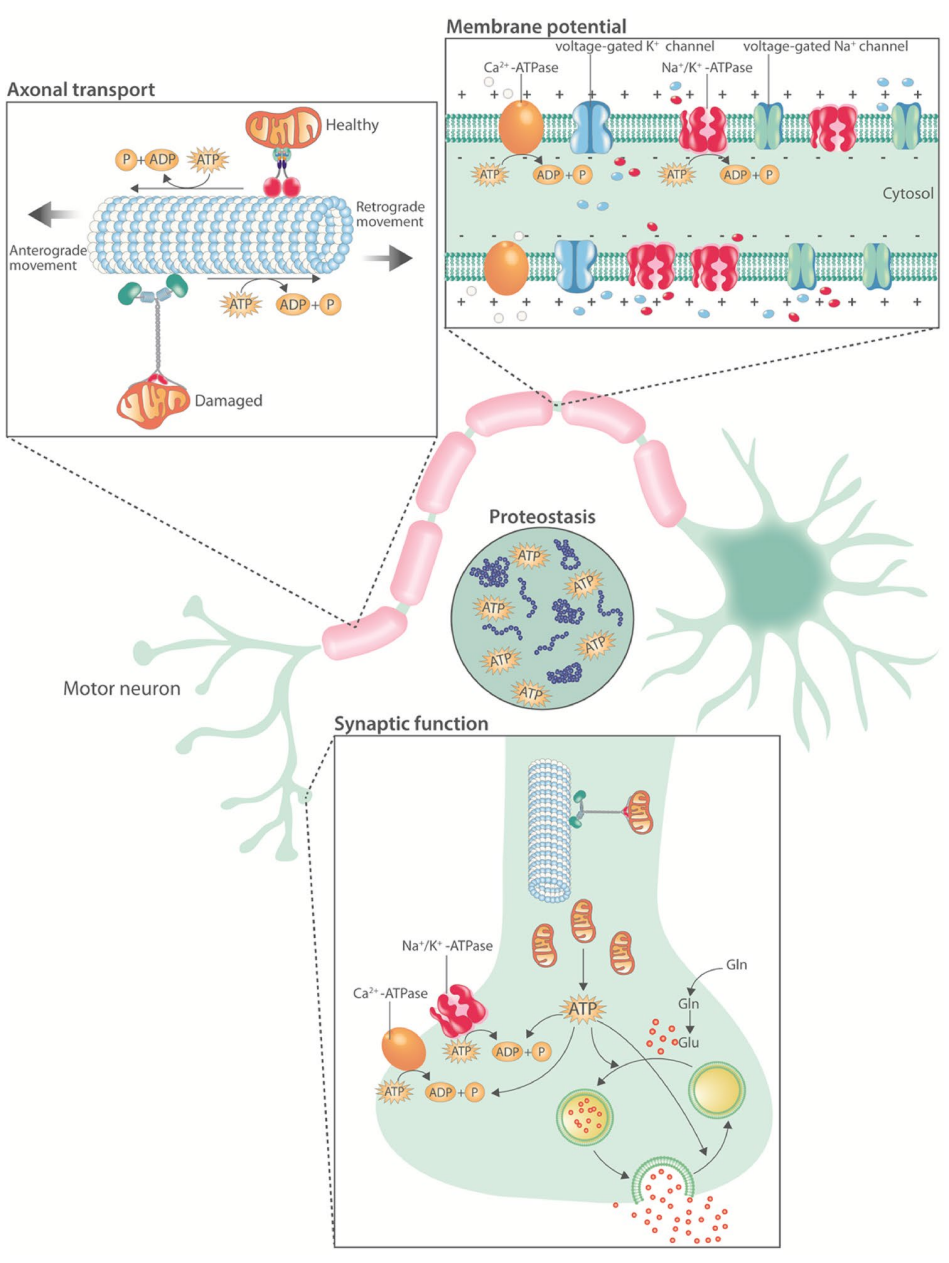
Overview of ATP consuming processes in motor neurons. Motor neuron physiology is highly energy demanding. First, the Na^+^/K^+^-ATPase and the Ca^2+^-ATPase hydrolyze ATP to establish and maintain the membrane potential and calcium homeostasis, respectively. Second, the molecular motors driving axonal transport depend on ATP hydrolysis. Third, synaptic activity is energetically expensive due to ion pumping, vesicular neurotransmitter uptake, and the endocytosis of vesicles from the synaptic cleft. Fourth, millimolar concentrations of ATP are required to maintain proteostasis. *Gln* glutamine, *Glu* glutamate, *ATP* adenosine triphosphate, *ADP* adenosine diphosphate, *P* inorganic phosphate

Since its first description by Charcot in 1869, the characteristic selective degeneration and death of motor neurons in ALS has remained an enigma. Neurons are large, polarized, excitable cells and, therefore, face unique challenges to maintain energy homeostasis (Fig. 1). They are the main contributors to the impressive energy demand of the central nervous system (CNS). First, action potential propagation is highly dependent on the Na^+^/K^+^-ATPase [75]. Second, due to the extensive length of their neurites, neurons, and, a fortiori, motor neurons, depend on axonal transport [155]. Importantly, the molecular motors driving axonal transport hydrolyze one ATP molecule, generated via on-board glycolysis [217], for every 8-nm displacement of their cargo [79, 167]. Since synapses are major sites of neuronal energy consumption, the trafficking of mitochondria is critical to meet synaptic energy requirements [174]. On top of this, high ATP concentrations are needed to keep proteins soluble [143]. The high dependence of motor neurons on continuous energy provision to maintain their normal function and integrity renders them particularly vulnerable to energetic stress [106].

To meet its substantial energy demand, the CNS largely relies on glucose as an energy substrate [121]. Recent in vitro and ex vivo studies have indicated though that neurons can readily oxidize several non-glucose substrates and that a switch towards glutamate oxidation could protect neurons from excitotoxic cell death [49, 61]. These data, nonetheless, require in vivo confirmation, since the absence of the blood–brain/spinal cord barrier and specific conditions of the CNS microenvironment might make it difficult to translate in vitro findings to an in vivo situation. Indeed, to date, the evidence indicates that only ketone bodies can sustain the energetic requirements of the CNS in conditions of severe glucose deprivation [35, 104]. Fatty acids are only poorly used as an energy substrate presumably due to: the slow passage of fatty acids across the blood–brain and blood–spinal cord barrier, the higher oxygen cost of fatty acid oxidation, the elevated superoxide generation during fatty acid oxidation in combination with poor anti-oxidant defense mechanisms of neurons, and the slower rate of ATP generation of fatty acid oxidation [168]. More than 90% of ATP generation in the CNS occurs via mitochondrial oxidative phosphorylation [82]. Acute fluctuations in ATP demand in the CNS are met by the creatine/phosphocreatine system, which represents an instant way to liberate high-energy phosphates for ATP by the transphosphorylation of phosphocreatine by creatine kinases [4]. Since ATP turnover in the CNS is high and substrate reserves small, the creatine/phosphocreatine system is crucial to buffer ATP fluctuations upon neuronal firing [16]. Moreover, the faster diffusion rate of phosphocreatine compared to ATP, and creatine compared to ADP [200], makes the creatine/phosphocreatine system suitable to connect sites of ATP generation to sites of ATP consumption.

**Fig. 2:**
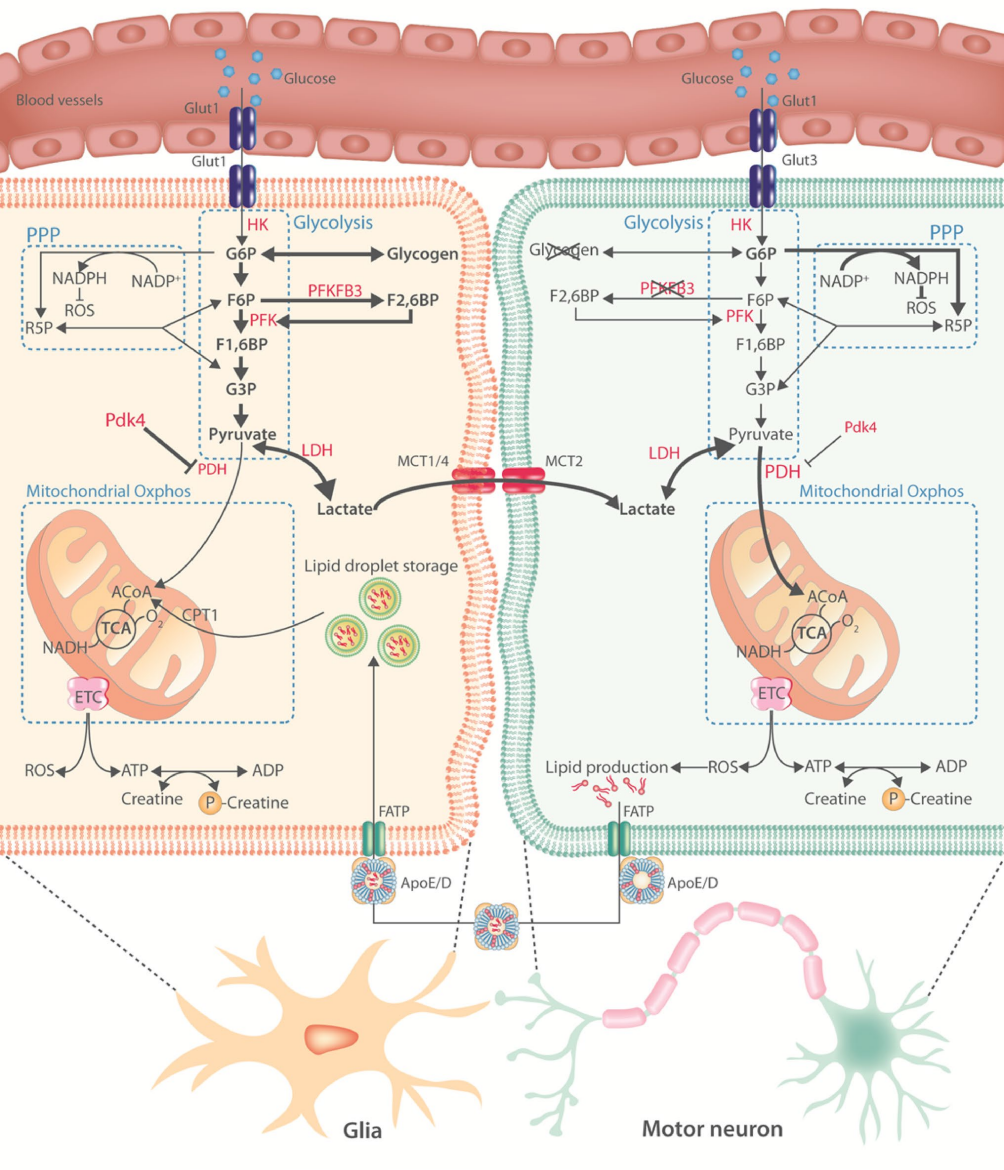
Motor neuron metabolism in health. An overview of the current knowledge on motor neuron energy metabolism. Metabolic pathways are indicated in blue, important enzymes in red. Bold black arrows indicate the main metabolic routes in glia or motor neurons. Neurons have low glycogen stores and low expression and activity of PFKFB3. Activity of PDH is higher in neurons compared to glia. These differences result in a predominantly oxidative versus glycolytic metabolic profile in neurons and glia, respectively. According to the astrocyte-neuronal lactate shuttle hypothesis, glia-derived lactate is shunted to motor neurons where it undergoes oxidative phosphorylation. ROS generation in motor neurons promotes lipid production. These lipids are transported to glia where they can be stored or catabolized. *Glut* glucose transporter, *HK* hexokinase, *G6P* glucose 6-phosphate, *R5P* ribose 5-phosphate, *F6P* fructose 6-phosphate, *PFK* phosphofructokinase, *PFKFB3* phosphofructokinase-2/fructose-2,6-bisphosphatase, *F1,6BP* fructose 1,6-bisphosphate, *NADP+* oxidized nicotinamide adenine dinucleotide phosphate, *NADPH* reduced nicotinamide adenine dinucleotide phosphate, *ROS* reactive oxygen species, *G3P* glyceraldehyde 3-phosphate, *LDH* lactate dehydrogenase, *PDH* pyruvate dehydrogenase, *Pdk4* pyruvate dehydrogenase kinase 4, *Oxphos* oxidative phosphorylation, *MCT* monocarboxylate transporter, *ACoA* acetyl coenzyme A, *CPT1* carnitine palmitoyltransferase 1, *TCA* tricarboxylic acid cycle, *O2* molecular oxygen, *NADH* reduced nicotinamide adenine dinucleotide, *ETC* electron transport chain, *FATP* fatty acid transport protein, *APOE/D* apolipoprotein E/D, *ATP* adenosine triphosphate, *ADP* adenosine diphosphate

Despite glucose being the dominant energy substrate, the CNS is a highly heterogeneous tissue composed of different cell types which show distinct metabolic profiles (Fig. 2). Differences are mainly studied in neurons and astrocytes, and indicate that neurons are predominantly oxidative and that astrocytes are predominantly glycolytic [22, 121, 219]. Under normal conditions, carbohydrate catabolism comprises the conversion of glucose to pyruvate via glycolysis followed by the full oxidation of glucose, or its metabolites pyruvate or lactate, in the mitochondria by the tricarboxylic acid (TCA) cycle and electron transport chain. Oxidative catabolism requires oxygen and generates 31–36 molecules of ATP for every molecule of glucose (or half of it if lactate or pyruvate is used as substrate). However, when the availability of oxygen is low (or in specific cell types—see below), glucose is only glycolytically catabolized to pyruvate, which generates only two molecules of ATP for each molecule of glucose, and is subsequently converted to lactate. This is a necessary step, since the regeneration of nicotinamide–adenine dinucleotide (NAD^+^) is required to keep glycolysis going when oxygen is limited [116]. Pyruvate dehydrogenase (PDH) is crucial to allow pyruvate entry into the TCA cycle and, hence, controls oxidative versus anaerobic catabolism. In astrocytes, PDH activity is low compared to neurons [73]; and pyruvate dehydrogenase kinase 4 expression, the main kinase suppressing PDH activity, is high, leading to higher glycolysis and lactate production [219]. In contrast, neurons have a lower rate of glycolysis under normal conditions due to the constant degradation of 6-phosphofructo-2-kinase/fructose-2,6-bisphosphatase 3 (PFKFB3), a key positive modulator of glycolysis, by the E3 ubiquitin ligase anaphase-promoting complex/cyclosome [18, 78]. In agreement, glia transport and metabolize glucose analogues faster than neurons both ex vivo [9] and in vivo [85]. In addition, a part of the glucose that is taken up by neurons does not enter glycolysis but is instead directed to the pentose phosphate pathway (PPP) during which the anti-oxidant reduced glutathione is regenerated (Fig. 2). Both overexpression [78] and stabilization [158] of PFKFB3 in neurons activated glycolysis at the expense of the PPP and resulted in oxidative stress and apoptotic death. These data suggest that neuronal homeostasis is particularly dependent on a tight balance between glycolysis and PPP flux to ensure sufficient ATP production while maintaining anti-oxidant status.

The more oxidative profile of neurons and more glycolytic profile of glia becomes more pronounced upon neuronal activity [76, 124], suggesting that other energy substrates are used to meet the neuronal energy demand during neuronal activity. Lactate is consumed in an activity-dependent manner in the CNS [162, 212] and is mainly oxidized by neurons compared to astrocytes [20, 196]. According to the astrocyte-neuronal lactate shuttle hypothesis, lactate is provided to neurons by astrocytes [147]. In brief, the reuptake of glutamate by astrocytes depletes their ATP stores, which stimulates the uptake of glucose and subsequently the glycolytic flux. The resulting lactate is mainly exported through the astrocyte-specific monocarboxylate transporter 4 (MCT4) and taken up by the neuron-specific MCT2 transporters. Next, it is fully oxidized to generate ATP (for a review, see [121]). In addition to lactate, astrocytes can also provide pyruvate and ketone bodies to the neurons [148]. Oxidative phosphorylation of glia-derived substrates in neurons leads to the generation of reactive oxygen species (ROS) which promote lipid production [112, 113]. Those lipids are transported to astrocytes via a fatty acid transport proteins (FATP) and apolipoprotein-dependent mechanism where they can form lipid droplets, be shunted into the ketogenic pathway, or undergo fatty acid oxidation [71] (Fig. 2). Impaired transport of lipids from neurons to glia accelerates neurodegeneration [112], suggesting a pro-survival function of neuron-derived lipids in glia by serving as in situ energy substrates under stress. Of note, while astrocyte-neuron metabolic coupling seems to be essential for nervous system homeostasis in *Drosophila* [204] and mice [64, 109, 120], it does not imply a complete metabolic compartmentalization of glycolysis versus oxidative phosphorylation in glia and neurons, respectively [7, 48]. Indeed, neurons also take up and metabolize glucose and increase glucose consumption in an activity-dependent manner [6, 47, 144]. In addition, neurons can catabolize glucose and lactate at the same time [115]. Therefore, it is likely that both oxidative phosphorylation of glia-derived energy substrates as well as neuronal glycolysis contribute to ATP production in high-energy demanding cellular situations.

Astrocyte contact with neurons is generally limited to the neuronal soma, synapses, and nodes of Ranvier, leaving the largest part of the axon without metabolic support from astrocytes. This is especially true for motor neurons. In contrast, oligodendrocytes are well connected to the axon and perfectly positioned to support the metabolic demands of neurons [151]. These glial cells highly express MCT1, which is the MCT with the highest affinity for lactate [152]. MCT1 inhibition in organotypic spinal cord slice cultures reduced motor neuron survival, but this effect was rescued by the addition of high concentrations of lactate to the culture medium [109], suggesting that oligodendrocyte-derived lactate contributes to the survival of motor neurons. In addition, astrocyte-to-oligodendrocyte coupling is essential for myelination [193]. Whether coupling between different glial cells is also involved in the metabolic support of motor neurons is unknown.

In summary, motor neurons require vast amounts of energy while having limited energy stores. Therefore, neuronal function and survival requires the continuous provision of substantial amounts of nutrients for ATP production. Under normal conditions, neurons are predominantly oxidative and astrocytes are predominantly glycolytic [219]. In addition, neurons keep a tight balance between glycolysis and flux through the PPP to maintain their anti-oxidant status while ensuring optimal ATP production. Of note, the metabolic characteristics of neurons have been studied to a large extent in cortical neurons. Whether and how motor neurons, due to their specific anatomy and microenvironment, have different metabolic properties, remain to be determined. Their high need for continuous energy provision, nonetheless, renders motor neurons particularly vulnerable to energetic stress [106]; and this could contribute to the selective vulnerability and degeneration of motor neurons observed in ALS. Indeed, fast-fatigable motor neurons, which have the highest peak needs of ATP [106], are initially targeted and are more severely affected during ALS compared to slow motor neurons [137].

## Motor neuron metabolism in ALS

### Cellular energy homeostasis is impaired in ALS

Mammalian AMP-activated protein kinase (AMPK) is a major cellular energy sensor activated by falling energy status. Upon activation, AMPK restores energy homeostasis by promoting catabolic pathways, resulting in ATP generation, and inhibiting anabolic pathways that consume ATP [74]. Enhanced AMPK activation was observed in motor neurons of ALS patients and correlated closely with the extent of cytoplasmic mislocalization of TDP-43 [114]. In NSC34 motor neuron-like cells, 5-aminoimidazole-4-carboxamide-1-β-*d*-ribofuranoside (AICAR)-mediated activation of AMPK caused TDP-43 mislocalization [114]. These data link energy depletion in human motor neurons to ALS-related TDP-43 pathology. AMPK activation was also increased in spinal cord cultures or lysates of mutant SOD1^G93A^ mice [110]. Pharmacological activation of AMPK worsened disease outcome in these mice [91]. In accordance, reducing AMPK-activity improved disease outcome in vitro or in *C. elegans* models expressing mutant SOD1 or TDP-43 [110]. These studies collectively show disturbed energy homeostasis at the cellular level in ALS and demonstrate its role in TDP-43 proteinopathy, the histopathological signature of ALS. While a clear mechanistic link between AMPK activation and TDP-43 mislocalization is currently lacking, nucleocytoplasmic transport is known to be an energy-dependent process [17]. It is, therefore, possible that cytoplasmic mislocalization of an aggregation prone protein such as TDP-43 [88] results from AMPK-mediated inhibition of nucleocytoplasmic transport.

### Mitochondrial dysfunction, an ALS hallmark

Mitochondrial dysfunction is a clinical hallmark of both sporadic and familial ALS [23, 55, 163]. As a consequence, multiple processes in which mitochondria play a key role are extensively investigated in ALS [182]. Seminal studies have shown dense clusters of mitochondria in the anterior horn of the lumbar spinal cord [164] and presynaptic mitochondrial swelling in motor neurons [180] of ALS patients. In addition, the amount of mitochondrial DNA, a direct marker of mitochondrial abundance, was reduced in the spinal cord from familial and sporadic ALS patients [207]. In mice carrying the SOD1^G37R^ mutation, membrane-bound vacuoles derived from degenerating mitochondria were observed in neurites [210]. Massive mitochondrial degeneration in motor neurons of mutant SOD1^G93A^ mice was already observed at disease onset [41, 102]. The observation that mitochondrial morphology is also abnormal in various murine FUS [80, 183] and TDP-43 models [170, 213] is important, since overexpression of human SOD1 per se, rather than the pathogenic effect of the mutation, induces mitochondrial vacuolization [84]. Besides morphological abnormalities, functional changes are present in ALS mitochondria. In spinal cord mitochondria from ALS patients, there was decreased activity of the electron transport chain (ETC) complexes I + III, II + III, and IV [207]. Decreased activity of the mitochondrial enzymes citrate synthase and cytochrome c oxidase was also reported in motor neurons from ALS patients [19]. Furthermore, impaired activities of complex I + III, II + III, and IV were also observed in mutant SOD1^G93A^ mice [129]. Importantly, reduced respiration and ATP synthesis preceded behavioral deficits in mutant SOD1^G93A^ mice [90, 129, 188], indicating a role in pathology. This has not yet been validated in other ALS models.

In addition to abnormal mitochondrial morphology and function, the cellular distribution of mitochondria is altered in ALS. In ALS patients, mitochondrial accumulation was observed in the cell body and proximal axon hillock [163]. Disturbed mitochondrial dynamics were also observed in embryonic and adult motor neurons expressing mutant SOD1^G93A^ [15, 45, 185], mutant TDP-43 overexpressing mice [122], and FUS patient-derived motor neurons [70]. Expressing mutant TDP-43 in motor neurons also induced aberrant mitochondrial distribution [205]. Miro1, a mitochondrial outer membrane protein coupling mitochondria to the axonal transport machinery, is downregulated in ALS, suggesting a mechanistic basis for impaired mitochondrial distribution in ALS [134, 218].

**Fig. 3:**
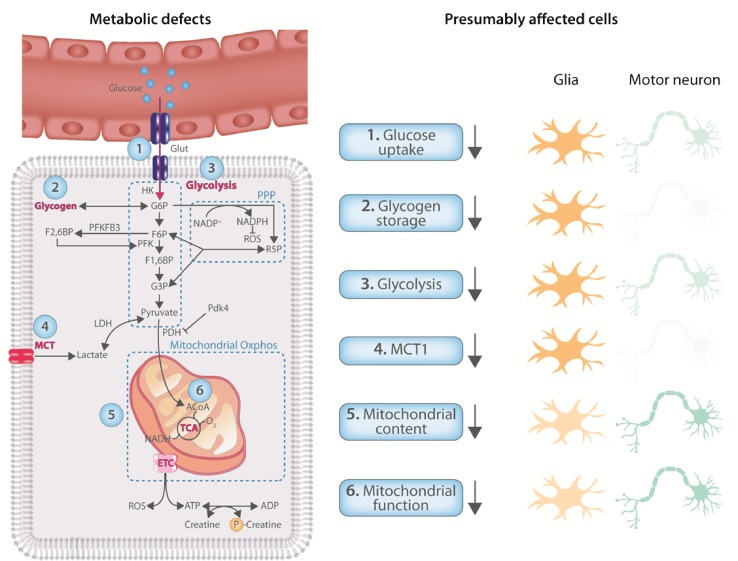
CNS energy metabolism is dysregulated in ALS. Metabolic processes shown to be affected in the CNS of ALS patients and/or models. Although most defects have not been attributed to a specific cell type, they are likely to result from either glia or motor neurons, or both. On the right, the presumably affected cell type(/s) is(/are) colored darker

Abnormal mitochondrial physiology is a consistent observation in ALS patients and in multiple ALS models (Fig. 3). In mutant SOD1 [90, 129, 188] and FUS [183] ALS mouse models, mitochondrial dysfunction is an early event and overexpressing peroxisome proliferator activated receptor-gamma coactivator 1 alpha (PGC1α), a major regulator of mitochondrial biogenesis, improved survival, motor function, and motor neuron survival in mutant SOD1^G93A^ mice [221]. PGC1α expression is also downregulated in the CNS of FUS-ALS mice and FUS patient derived motor neurons [10]. Therefore, improving mitochondrial biogenesis may be an attractive therapeutic strategy for ALS. It should be noted that mitochondrial abnormalities in ALS are not restricted to motor neurons. In skeletal muscle from patients, mitochondria are also structurally [130, 203] and functionally [38, 58] abnormal. While alterations in skeletal muscle can affect neuromuscular junction integrity [209], their role in ALS remains controversial (for a review, see [117]).

This said, the underlying cause of mitochondrial dysfunction in ALS and whether mitochondrial dysfunction is causally linked to motor neuron pathology in ALS still remains an open question. It is also not yet clear whether mitochondrial defects are present in all ALS subtypes. Moreover, mechanisms linking mutations in TDP-43 and FUS to aberrant mitochondrial physiology remain to be determined. Overall, the current literature suggests that aberrant mitochondrial physiology could be an important contributing factor to the pathogenesis of ALS.

### Carbohydrate metabolism and ALS

In line with mitochondrial dysfunction, glucose uptake in the motor-sensory cortex of ALS patients is reduced [77, 140]. FDG-PET studies linked the reduction of glucose uptake and phosphorylation to the severity of the disease [42]. Another FDG-PET study consisting of 81 patients with a suspected diagnosis of ALS was able to correctly classify 95% of ALS cases, indicating reduced glucose uptake as an early diagnostic event in ALS [197]. In mutant SOD1^G93A^ mice, glucose uptake in the spinal cord increased pre-symptomatically, but declined progressively during disease progression [133]. Under physiological conditions, there is a tight coupling between blood flow and glucose metabolism in the CNS [66]. Remarkably, the pre-symptomatic increase in glucose uptake in the spinal cord of mutant SOD1^G93A^ mice was not matched by increases in spinal blood flow [133]. Despite reduced glucose uptake, the spinal cord from end-stage mutant SOD1^G93A^ mice, as well as from autopsied ALS patients, is characterized by elevated concentrations of glycogen [50]. Blood flow–metabolism uncoupling together with increased glycogen storage in the CNS suggests a decreased ability to catabolize carbohydrates in mutant SOD1^G93A^ mice. However, it is debated whether reduced glucose uptake in the CNS of ALS patients reflects a reduction in neuronal carbohydrate catabolism or a reduction in the number of motor neurons. Nevertheless, the ability to catabolize carbohydrates appears to be reduced in human ALS patients as the expression of phosphoglucomutase 2 like 1 and phosphoglycerate kinase, two key enzymes in glycolysis, is downregulated in fibroblasts from sporadic ALS patients [157]. In agreement, a recent proteomic study in sporadic ALS skin fibroblasts showed a marked reduction in components of glycolysis [188]. Whole-genome expression profiling in the motor cortex of sporadic ALS patients also showed a significant downregulation of glycolytic genes [108]. Another study in *post-mortem* cortex of ALS patients identified an over twofold reduction in PFKFB3 mRNA content [206]. In contrast, introducing mutant SOD1 in human fibroblasts or NSC34 motor neuron-like cells increased glycolysis and reduced mitochondrial ATP generation [3, 195]. Given the limited capacity of neurons to upregulate glycolysis [78, 158], the physiological relevance to ALS of the upregulation of glycolysis in these cells remains to be established. Nevertheless, neurons can upregulate glycolysis [47] and oxidative stress is evident in *post*-*mortem* samples of ALS patients [63]. It is, therefore, possible that during severe energetic stress, motor neurons sacrifice their redox status to alleviate energetic stress and eventually die due to excessive oxidative stress.

It remains to be determined whether these metabolic alterations are taking place in motor neurons or glia (Fig. 3). Given that in both the cortex and the spinal cord, motor neurons represent a quantitatively minor population, functional metabolic studies on induced pluripotent stem cell (iPSC)-derived motor neurons from patients could provide valuable insight. Compared to neurons, glia are more glycolytic, and, therefore, likely significantly contribute to the observed reductions in transcription level of key glycolytic transcripts in the CNS of ALS patients. In oligodendrocytes from ALS patient and mutant SOD1^G93A^ mice, the expression of MCT1 transporters is downregulated [92, 109, 150]. In addition, the downregulation of the glutamate transporter GLT-1 in astrocytes from ALS patients is well established [161]. These observations suggest that the reduced glycolytic capacity is, at least in part, due to changes in glial metabolism.

Taken together, reductions in glucose uptake and increased glycogen storage in the CNS of ALS patients and ALS animal models suggest a reduced capacity to catabolize glucose. Noteworthy, riluzole enhanced CNS glucose uptake both in vitro [43] and in vivo [32], suggesting that improving glucose transport rates in ALS affected cells may be a potential therapeutic avenue. While studies evaluating specific pathways are scarce, glycolysis seems to be downregulated. How different cell types in the CNS contribute to reduced carbohydrate catabolism remains to be investigated. Therefore, future studies investigating the metabolic fate of glucose, using, i.e., traceable glucose analogues [8], in ALS models are urgently needed. Moreover, it is not clear how reduced carbohydrate catabolism might affect neuronal function.

## Metabolic treatments tested in ALS

Energy metabolism is altered in ALS (Fig. 3) and correlates to disease progression, suggesting a role for energy metabolism in ALS pathogenesis. As a consequence, targeting metabolism represents a rational strategy to treat ALS. Below, we will give an overview of therapeutic approaches that target energy metabolism and have been tested in ALS patients and/or preclinical models (for an overview, see Table 1). Most approaches focus on increasing the provision of energetic substrates or improving mitochondrial function. Some strategies target the electron transport chain as the most important cellular source of oxidative stress. Creatine has also been investigated for its energy buffering capacities. Of note, most metabolic treatments have multiple mechanisms of action. One example is dichloroacetate, which improves mitochondrial function indirectly by stimulating the conversion of pyruvate to acetyl coenzyme A (ACoA), and, therefore, also provides additional energy substrates to the TCA cycle. For reasons of clarity, we classified treatments according to their principal mechanism of action.

**Table 1:** Metabolic treatments tested in ALS

Putative mechanism of action	Metabolic treatment	Effect on ALS models	Effect on ALS patients
Energy buffering and transport	Creatine	Improved lifespan, motor neuron survival, and motor function in mutant SOD1^G93A^ mice [87]	No efficacy in phase II/III clinical trials [55, 139, 151]
Oxidative stress	Coenzyme Q10	Improved survival in mutant SOD1^G93A^ mice—[111]	No efficacy in phase II clinical trial [81]
	MitoQ	Reduced toxicity of mutant SOD1^G93A^ rat astrocytes to healthy motor neurons in co-culture [24] Improved motor function, survival, and histopathology in mutant SOD1^G93A^ mice [114]	To be tested
	Dexpramipexole	Improved survival, and motor function in mutant SOD1^G93A^ mice in one study [36], but not in a second study [177] No effect in patient derived iPSCs [189] No effect in rat cortical neurons transfected with mutant or wild-type TDP-43 [177]	No efficacy in phase III clinical trial [33]
	Edaravone	Delayed motor neuron degeneration and spinal cord SOD1 deposition in mutant SOD1^G93A^ mice [71] Delayed disease progression in wobbler mice [69] Improved motor performance in mutant SOD1^H46R^ rats [5]	Efficacy in a subset of ALS patients [184], FDA-approved
Additional and/or alternative fuel	High caloric diet	Delayed disease onset and extended survival in mutant SOD1^G93A^, mutant SOD1^G86R^, and mutant TDP-43^A315T^ mice [30, 46] Delayed motor neuron loss in the spinal cord of mutant SOD1^G93A^ mice [46]	Promising results in a phase II clinical trial [182]
	Ketone bodies	Ketogenic diets delay disease onset, improved motor neuron survival but not lifespan in mutant SOD1^G93A^ mice [195] Ketone esters are to be tested in ALS models	To be tested
	Medium-chain triglycerides	Delayed disease onset, and improved motor neuron survival in mutant SOD1^G93A^ mice [167, 193]	To be tested
	Pyruvate	Improved motor performance, disease progression, and lifespan in mutant SOD1^G93A^ mice [121] but not in a subsequent study [48]	To be tested
Mitochondrial function	Dichloroacetate	Improved survival, delayed disease onset, and improved motor neuron survival in mutant SOD1^G93A^ mice [113, 120]	To be tested
	Acetyl-l-carnitine	Neurotrophic effects in rat embryonic motor neurons [10] Improved survival in mutant SOD1^G93A^ mice [86]	Promising results in a phase II clinical trial [8]

### Creatine

As described above, the creatine/phosphocreatine system plays a crucial role in neurons for cellular energy buffering and transport [4]. In mutant SOD1^G93A^ mice, creatine treatment prevented ATP depletion in the cerebellar cortex and spinal cord [26], but not in skeletal muscle [46]. Creatine treatment in mutant SOD1^G93A^ mice markedly improved motor neuron survival but only moderately enhanced motor function and lifespan [101]. No studies evaluated the effect of creatine in other ALS mouse models. In ALS patients, creatine supplementation did not affect survival or disease progression [69, 160, 173]. Several reasons can explain the different effect of creatine in mutant SOD1^G93A^ mice and ALS patients. First, the efficacy of creatine in mutant SOD1^G93A^ mice can, at least in part, be explained by a buffering effect of creatine on SOD1 overexpression-related mitochondriopathy [13, 84]. In addition, the treatment was initiated at different disease stages in mice compared to patients. While creatine intake in mutant SOD1^G93A^ mice was started before symptom onset and before the reduction in ATP concentration in the CNS [26], ALS patients’ treatment started at least 1 year after symptom onset when already extensive alterations in energy metabolism are present. While creatine supplementation is able to prevent neuronal ATP depletion in some conditions and for short periods of time [186], it acts by energy buffering and transport without contributing to ATP production [4]. Therefore, it is likely that when treatment only commences during progressed disease states, the cascade of metabolic dysfunction is too far advanced for interventions to be successful.

### Targeting oxidative stress

The interest in the role of oxidative stress was nurtured for decades by the finding that mutations in *SOD1* can cause ALS [159]. Whether oxidative stress is a primary or secondary disease mechanism in human ALS is still unclear. The recent discovery that the free radical scavenger edaravone improves ALS functional rating scale (ALSFRS) scores of a subgroup of ALS patients suggests that oxidative stress affects motor neuron death [211]. Nevertheless, most clinical trials targeting oxidative stress failed to demonstrate clinical efficacy (see below). Given the vulnerability of motor neurons to oxidative stress [172], neurons employ different strategies to minimize ROS accumulation [168]. Hence, oxidative stress might be an indicator of advanced cellular damage rather than an early pathological event. Identifying the exact underlying mechanism responsible for the efficacy of edaravone in ALS patient subpopulations could provide further insight in the role of oxidative stress in ALS.

#### Coenzyme Q10 and MitoQ

Coenzyme Q, also known as ubiquinone, is the only endogenous lipid-soluble anti-oxidant found in humans. It acts as an essential cofactor in the electron transport chain where it accepts electrons from complex I and II and shuttles them to complex III. Its quinone group can be reduced to quinol, explaining its anti-oxidative properties [37]. In mutant SOD1^G93A^ mice, coenzyme Q10 induced a mild improvement in survival [128]. However, a phase II clinical trial, treating ALS patients with small amounts of coenzyme Q10 for 9 months, did not observe improvements on ALSFRS [95]. Of note, the feeding regimen in this study only induced a moderate increase in plasma coenzyme Q10 levels and the previous studies in Parkinson’s disease suggest that doses up to 100-fold of the dose used are needed to slow disease progression [178, 179]. Although administration of high doses of exogenous coenzyme Q10 is well tolerated in humans [178], its hydrophobicity compromises bioavailability [128]. To improve this, MitoQ, a mitochondrion-targeted and recyclable coenzyme Q10 analogue, was developed [96]. MitoQ showed enhanced bioavailability and improved mitochondrial function in different neuronal cell types exposed to oxidative stress [189]. Rat mutant SOD1^G93A^ astrocytes were previously shown to be toxic to wild-type motor neurons [72, 214]. Interestingly, pretreatment of mutant SOD1^G93A^ astrocytes with low doses of MitoQ reduced oxidative damage and enhanced mitochondrial ATP generation in motor neurons [29]. Adding MitoQ to the drinking water of mutant SOD1^G93A^ mice slowed the decline of mitochondrial function in spinal cord and muscle, reduced spinal cord oxidative damage, improved the integrity of neuromuscular junctions, increased hindlimb strength, and prolonged the life span of mutant SOD1^G93A^ mice [132]. While these results are promising, no clinical trials assessed the efficacy or tolerability of MitoQ in ALS patients thus far.

#### Dexpramipexole

Pramipexole is a dopamine agonist approved to treat Parkinson’s disease [171] and restless leg syndrome [125]. In addition, pramipexole demonstrates anti-oxidative properties [107]. Dexpramipexole, the *R*+ enantiomer of pramipexole, has a 100-fold lower affinity for dopamine receptors than pramipexole, but is equipotent to scavenge ROS [44, 68]. Dexpramipexole improved metabolic efficiency, defined as the amount of ATP generated for a given value of oxygen consumption, in whole rat brain-derived mitochondria [2]. In mutant SOD1^G93A^ mice, dexpramipexole prolonged survival and delayed motor deterioration [44]. In a phase II clinical trial, dexpramipexole administration to ALS patients was well tolerated and tended to attenuate functional decline in a dose-dependent manner [39]. However, dexpramipexole did not differ from placebo for any efficacy measurement in a subsequent phase III clinical trial [40]. Moreover, dexpramipexole did not show a protective effect in subsequent preclinical studies in mutant SOD1^G93A^ mice [202], ALS patient derived iPSCs [216], or rat cortical neurons transfected with mutant or wild-type human TDP-43 [202].

### Fueling energy metabolism

Imbalanced energy homeostasis is an early and persistent observation throughout the course of ALS. Moreover, endogenous energy stores, which are mainly located in skeletal muscle and adipose tissue, are progressively depleted during disease progression. Providing additional energetic substrates may, therefore, improve the clinical outcome. In addition, impairments in carbohydrate metabolism suggest that energy substrates other than glucose might have more pronounced effects. Enhancing the availability of specific metabolites is an alternative way to improve ATP production. This could be particularly important in neurons to compensate for losses of the TCA intermediate α-ketoglutarate that occur through the release of the α-ketoglutarate-derived neurotransmitters glutamate and GABA [169].

#### High caloric diets to treat ALS

In mutant SOD1^G93A^, SOD1^G86R^, and TDP43^A315T^ overexpressing mice [36, 56], a high fat diet delayed disease onset and extended survival, while caloric restriction shortened the lifespan of mutant SOD1^G93A^ mice [145, 146]. Moreover, a high fat diet attenuated motor neuron loss in the spinal cord of mutant SOD1^G93A^ mice [56]. A small prospective study in ALS patients showed that high caloric diets were able to abolish weight loss [51]. In a phase II clinical trial involving 20 patients, subjects were assigned to one of three diets using gastrostomy: caloric intake designed to match caloric expenditure, a high fat diet, or a high carbohydrate diet both providing an excess of calories. One out of 17 patients assigned to a hypercaloric diet died during the 5-month follow-up period compared with three out of seven patients assigned to the control group [208]. While this study was promising, a sufficiently powered phase III clinical trial to examine the effect of hypercaloric diets on survival and functional outcome in ALS patients is still lacking. Moreover, whether the composition of the hypercaloric diet matters is an outstanding question.

#### Ketone bodies, ketogenic diets, and beyond

Ketone bodies are energy substrates endogenously produced from fat when glucose availability is limited. While the liver is the major site of ketogenesis, glial cells are also able to produce ketone bodies [5]. Ketone bodies have a high metabolic efficiency generating 30% more energy per molecule oxygen than pyruvate [199]. Therefore, ketones are suited to meet high-energy demands. Besides being an energy substrate, ketone bodies are signaling metabolites acting as histone deacetylase inhibitors to reduce oxidative stress [177]. In mutant SOD1^G93A^ mice, a ketogenic diet delayed disease onset and improved motor neuron survival without affecting lifespan [222]. However, as ketogenic diets are associated with a loss of muscle mass [24], the potential beneficial effect of ketosis on lifespan may be blunted. Recently, ketone esters have emerged as a novel approach to raise blood ketone bodies immediately [34, 97], even when co-ingesting high amounts of carbohydrates and proteins [198]. Ketone esters have improved disease outcome in an Alzheimer’s disease mouse model [93]. In ALS, the therapeutic potential of ketone esters is unexplored.

#### Medium-chain triglycerides

Medium-chain triglycerides were previously used as a more palatable alternative to ketogenic diets to treat epilepsy and Alzheimer’s disease [181]. Medium-chain triglycerides are able to cross the blood–brain barrier via diffusion and enter neurons via monocarboxylate transporters [168] where they can undergo β-oxidation to form ACoA and ketone bodies, which fuel the TCA cycle [192]. Two medium-chain triglycerides investigated in the context of ALS are caprylic triglyceride and triheptanoin, the triglycerides of octanoic acid (8C fatty acid) and heptanoic acid (7C fatty acid), respectively. Early administration of caprylic triglyceride to mutant SOD1^G93A^ mice delayed disease onset, improved motor performance, reduced motor neuron loss, and promoted mitochondrial oxygen consumption in the spinal cord [220]. Pre-symptomatic triheptanoin ingestion also delayed disease onset and reduced motor neuron loss at symptom onset in mutant SOD1^G93A^ mice [191]. Clinical trials evaluating safety or efficacy of medium-chain triglyceride treatments in ALS patients are lacking.

#### Pyruvate

Pyruvate is the end product of glycolysis and represents a mitochondrial fuel entering the TCA cycle after conversion to ACoA. Pyruvate is neuroprotective in models for epilepsy [153] and Alzheimer’s disease [83]. The neuroprotective properties of pyruvate are multifaceted and originate from anti-oxidant properties, the ability to facilitate glutamate efflux from the brain, anti-inflammatory effects, and their ability to increase TCA cycling [223]. In the context of ALS, administration of 1 g pyruvate/kg body weight/week to mutant SOD1^G93A^ mice prolonged the lifespan by 12 days, slowed disease progression, and improved motor performance when starting the treatment at the age of 70 days [142]. However, another study in which mutant SOD1^G93A^ mice received 0.5 g/kg body weight six times a week starting from the same age did not improve survival or rotarod performance [59]. There are no clinical trials available assessing the effect of pyruvate intake in ALS patients.

### Enhancing mitochondrial function

In the presence of decreased mitochondrial function, providing additional and/or alternative energy substrates may be insufficient. Enhancing mitochondrial function and hence metabolic efficiency may be necessary.

#### Dichloroacetate

Increasing the conversion of pyruvate into ACoA, by providing the pyruvate dehydrogenase kinase inhibitor dichloroacetate improved survival, delayed disease onset, and reduced spinal motor neuron loss in mutant SOD1^G93A^ mice [131, 141]. As dichloroacetate blunted the reduction of expression of mitochondrial genes seen in mutant SOD1^G93A^ skeletal muscle during disease progression, improving mitochondrial function could elicit this effect [141]. While in NSC34 motor neuron-like cells mutant SOD1 increased pyruvate dehydrogenase kinase expression and lactate production [195], it was not tested if dichloroacetate treatment improved mitochondrial function in the CNS of mutant SOD1^G93A^ mice. Although doses of dichloroacetate used in preclinical ALS studies are well tolerated in patients with advanced solid tumors [33], ALS clinical trials are lacking.

#### Acetyl-l-carnitine

Acetyl-l-carnitine is the acetyl-ester of l-carnitine. Acetyl-l-carnitine is an important cellular source of acetyl groups to generate ACoA in high-energy demanding situations [139]. Acetyl-l-carnitine also mediates transport of long chain fatty acids across mitochondrial membranes and is, therefore, rate limiting for β-oxidation. While the neuroprotective effects of acetyl-l-carnitine are mainly described in cortical neurons [166, 175], an early study showed neuroprotective and neurotrophic effects of acetyl-l-carnitine in embryonic rat motor neurons [14]. Moreover, administration of l-carnitine to symptomatic mutant SOD1^G93A^ mice improved survival [100]. Based on these findings, a randomized double-blind placebo-controlled phase II trial was performed in 82 patients. Subjects ingested 3 g of acetyl-l-carnitine or placebo each day together with riluzole. Acetyl-l-carnitine was well tolerated, and respiratory capacity and ALSFRS showed mild improvements. In addition, median survival doubled in the acetyl-l-carnitine group compared to the placebo group [11]. Despite these results, a larger phase III trial has not yet been performed.

## Conclusion and future perspectives

While dysregulated systemic energy metabolism is now well established in ALS patients, energy metabolism has received a little attention in ALS research due to its association with mutant SOD1 models. It now becomes obvious that abnormal energy metabolism also has a role in more recently developed ALS models [118, 170, 183, 205]. In ALS motor neurons and glia, both mitochondrial and glycolytic energy metabolism seem to be impaired, but the molecular mechanisms underlying energetic stress remain unknown. Since motor neuron physiology is highly energy demanding, impairments in energy metabolism could, at least in part, explain the selective dying of motor neurons in ALS. As a consequence, targeting defects in energy metabolism in ALS represents a rational therapeutic strategy. Manipulating energy metabolism is a particularly potent strategy to treat complex diseases due to its intimate link to epigenetic control [60, 94, 119] and is, therefore, increasingly recognized as therapeutic target in cancer [28], immunodeficiency [123], and stroke [156]. To date, a unifying view on how different metabolic pathways converge and whether metabolic alterations contribute to disease etiology in ALS is non-existing. Future work using direct measurements of metabolic fluxes is clearly needed to obtain a more in-depth understanding of motor neuron metabolism in health and disease. Moreover, due to the compartmentalization of specific energy requiring processes in motor neurons, defining the role of metabolism and ALS-related motor neuron dysfunction requires high-resolution and spatial subdivision of metabolic and functional analyses. Knowing how ALS motor neurons differ metabolically from healthy motor neurons could offer the necessary insights to develop future therapeutic approaches in ALS. Another relevant area for future research is to explore the metabolic crosstalk between motor neurons and glial cells, as well as other disease-relevant cells such as the muscle.

In conclusion, it is too early to consider ALS at present as a metabolic disease. However, the massive amount circumstantial evidence linking energy metabolism with ALS pathophysiology underscores the therapeutic potential of targeting metabolism. As is the case for many other pathways and mechanisms proposed to play a crucial role in ALS, the ultimate proof that disturbances in metabolism are causally linked to the selective motor neuron death in ALS will be a positive clinical trial with a therapeutic strategy tackling energy metabolism in patients. In the meantime, we strongly believe that a better understanding of the metabolic biology of ALS could lead to the identification of novel therapeutic targets.

## References

[1] Ahmed RM, Irish M, Piguet O, Halliday GM, Ittner LM, Farooqi S, Hodges JR, Kiernan MC (2016). Amyotrophic lateral sclerosis and frontotemporal dementia: distinct and overlapping changes in eating behaviour and metabolism. Lancet Neurol.

[2] Alavian KN, Dworetzky SI, Bonanni L, Zhang P, Sacchetti S, Mariggio MA, Onofrj M, Thomas A, Li H, Mangold JE, Signore AP, Demarco U, Demady DR, Nabili P, Lazrove E, Smith PJ, Gribkoff VK, Jonas EA (2012). Effects of dexpramipexole on brain mitochondrial conductances and cellular bioenergetic efficiency. Brain Res.

[3] Allen SP, Rajan S, Duffy L, Mortiboys H, Higginbottom A, Grierson AJ, Shaw PJ (2014). Superoxide dismutase 1 mutation in a cellular model of amyotrophic lateral sclerosis shifts energy generation from oxidative phosphorylation to glycolysis. Neurobiol Aging.

[4] Andres RH, Ducray AD, Schlattner U, Wallimann T, Widmer HR (2008). Functions and effects of creatine in the central nervous system. Brain Res Bull.

[5] Auestad N, Korsak RA, Morrow JW, Edmond J (1991). Fatty acid oxidation and ketogenesis by astrocytes in primary culture. J Neurochem.

[6] Bak LK, Schousboe A, Sonnewald U, Waagepetersen HS (2006). Glucose is necessary to maintain neurotransmitter homeostasis during synaptic activity in cultured glutamatergic neurons. J Cereb Blood Flow Metab.

[7] Bak LK, Walls AB (2018). Crosstalk opposing view: lack of evidence supporting an astrocyte-to-neuron lactate shuttle coupling neuronal activity to glucose utilisation in the brain. J Physiol.

[8] Barros LF, Bolanos JP, Bonvento G, Bouzier-Sore AK, Brown A, Hirrlinger J, Kasparov S, Kirchhoff F, Murphy AN, Pellerin L, Robinson MB, Weber B (2017). Current technical approaches to brain energy metabolism. Glia.

[9] Barros LF, Courjaret R, Jakoby P, Loaiza A, Lohr C, Deitmer JW (2009). Preferential transport and metabolism of glucose in Bergmann glia over Purkinje cells: a multiphoton study of cerebellar slices. Glia.

[10] Bayer H, Lang K, Buck E, Higelin J, Barteczko L, Pasquarelli N, Sprissler J, Lucas T, Holzmann K, Demestre M, Lindenberg KS, Danzer KM, Boeckers T, Ludolph AC, Dupuis L, Weydt P, Witting A (2017). ALS-causing mutations differentially affect PGC-1alpha expression and function in the brain vs. peripheral tissues. Neurobiol Dis.

[11] Beghi E, Pupillo E, Bonito V, Buzzi P, Caponnetto C, Chio A, Corbo M, Giannini F, Inghilleri M, Bella VL, Logroscino G, Lorusso L, Lunetta C, Mazzini L, Messina P, Mora G, Perini M, Quadrelli ML, Silani V, Simone IL, Tremolizzo L, Italian ALSSG (2013). Randomized double-blind placebo-controlled trial of acetyl-l-carnitine for ALS. Amyotroph Lateral Scler Frontotemporal Degener.

[12] Bensimon G, Lacomblez L, Meininger V, ALS/Riluzole Study Group (1994). A controlled trial of riluzole in amyotrophic lateral sclerosis. N Engl J Med.

[13] Bergemalm D, Jonsson PA, Graffmo KS, Andersen PM, Brannstrom T, Rehnmark A, Marklund SL (2006). Overloading of stable and exclusion of unstable human superoxide dismutase-1 variants in mitochondria of murine amyotrophic lateral sclerosis models. J Neurosci.

[14] Bigini P, Larini S, Pasquali C, Muzio V, Mennini T (2002). Acetyl-l-carnitine shows neuroprotective and neurotrophic activity in primary culture of rat embryo motoneurons. Neurosci Lett.

[15] Bilsland LG, Sahai E, Kelly G, Golding M, Greensmith L, Schiavo G (2010). Deficits in axonal transport precede ALS symptoms in vivo. Proc Natl Acad Sci USA.

[16] Boero J, Qin W, Cheng J, Woolsey TA, Strauss AW, Khuchua Z (2003). Restricted neuronal expression of ubiquitous mitochondrial creatine kinase: changing patterns in development and with increased activity. Mol Cell Biochem.

[17] Boeynaems S, Bogaert E, Van Damme P, Van Den Bosch L (2016). Inside out: the role of nucleocytoplasmic transport in ALS and FTLD. Acta Neuropathol.

[18] Bolanos JP, Almeida A, Moncada S (2010). Glycolysis: a bioenergetic or a survival pathway?. Trends Biochem Sci.

[19] Borthwick GM, Johnson MA, Ince PG, Shaw PJ, Turnbull DM (1999). Mitochondrial enzyme activity in amyotrophic lateral sclerosis: implications for the role of mitochondria in neuronal cell death. Ann Neurol.

[20] Boumezbeur F, Petersen KF, Cline GW, Mason GF, Behar KL, Shulman GI, Rothman DL (2010). The contribution of blood lactate to brain energy metabolism in humans measured by dynamic C-13 nuclear magnetic resonance spectroscopy. J Neurosci.

[21] Bouteloup C, Desport JC, Clavelou P, Guy N, Derumeaux-Burel H, Ferrier A, Couratier P (2009). Hypermetabolism in ALS patients: an early and persistent phenomenon. J Neurol.

[22] Bouzier-Sore AK, Voisin P, Bouchaud V, Bezancon E, Franconi JM, Pellerin L (2006). Competition between glucose and lactate as oxidative energy substrates in both neurons and astrocytes: a comparative NMR study. Eur J Neurosci.

[23] Bowling AC, Schulz JB, Brown RH, Beal MF (1993). Superoxide dismutase activity, oxidative damage, and mitochondrial energy metabolism in familial and sporadic amyotrophic lateral sclerosis. J Neurochem.

[24] Bravata DM, Sanders L, Huang J, Krumholz HM, Olkin I, Gardner CD, Bravata DM (2003). Efficacy and safety of low-carbohydrate diets: a systematic review. JAMA.

[25] Brown RH, Al-Chalabi A (2017). Amyotrophic lateral sclerosis. N Engl J Med.

[26] Browne SE, Yang LC, DiMauro JP, Fuller SW, Licata SC, Beal MF (2006). Bioenergetic abnormalities in discrete cerebral motor pathways presage spinal cord pathology in the G93A SOD1 mouse model of ALS. Neurobiol Dis.

[27] Cai B, Zhu Y, Ma Y, Xu Z, Zao Y, Wang J, Lin Y, Comer GM (2003). Effect of supplementing a high-fat, low-carbohydrate enteral formula in COPD patients. Nutrition.

[28] Cantelmo AR, Conradi LC, Brajic A, Goveia J, Kalucka J, Pircher A, Chaturvedi P, Hol J, Thienpont B, Teuwen LA, Schoors S, Boeckx B, Vriens J, Kuchnio A, Veys K, Cruys B, Finotto L, Treps L, Stav-Noraas TE, Bifari F, Stapor P, Decimo I, Kampen K, De Bock K, Haraldsen G, Schoonjans L, Rabelink T, Eelen G, Ghesquiere B, Rehman J, Lambrechts D, Malik AB, Dewerchin M, Carmeliet P (2016). Inhibition of the glycolytic activator PFKFB3 in endothelium induces tumor vessel normalization, impairs metastasis, and improves chemotherapy. Cancer Cell.

[29] Cassina P, Cassina A, Pehar M, Castellanos R, Gandelman M, de Leon A, Robinson KM, Mason RP, Beckman JS, Barbeito L, Radi R (2008). Mitochondrial dysfunction in SOD1G93A-bearing astrocytes promotes motor neuron degeneration: prevention by mitochondrial-targeted antioxidants. J Neurosci.

[30] Chiang PM, Ling J, Jeong YH, Price DL, Aja SM, Wong PC (2010). Deletion of TDP-43 down-regulates Tbc1d1, a gene linked to obesity, and alters body fat metabolism. Proc Natl Acad Sci USA.

[31] Chio A, Calvo A, Ilardi A, Cavallo E, Moglia C, Mutani R, Palmo A, Galletti R, Marinou K, Papetti L, Mora G (2009). Lower serum lipid levels are related to respiratory impairment in patients with ALS. Neurology.

[32] Chowdhury GM, Banasr M, de Graaf RA, Rothman DL, Behar KL, Sanacora G (2008). Chronic riluzole treatment increases glucose metabolism in rat prefrontal cortex and hippocampus. J Cereb Blood Flow Metab.

[33] Chu QS, Sangha R, Spratlin J, Vos LJ, Mackey JR, McEwan AJ, Venner P, Michelakis ED (2015). A phase I open-labeled, single-arm, dose-escalation, study of dichloroacetate (DCA) in patients with advanced solid tumors. Investig New Drugs.

[34] Clarke K, Tchabanenko K, Pawlosky R, Carter E, Todd King M, Musa-Veloso K, Ho M, Roberts A, Robertson J, Vanitallie TB, Veech RL (2012). Kinetics, safety and tolerability of (*R*)-3-hydroxybutyl (*R*)-3-hydroxybutyrate in healthy adult subjects. Regul Toxicol Pharmacol.

[35] Cotter DG, d’Avignon DA, Wentz AE, Weber ML, Crawford PA (2011). Obligate role for ketone body oxidation in neonatal metabolic homeostasis. J Biol Chem.

[36] Coughlan KS, Halang L, Woods I, Prehn JH (2016). A high-fat jelly diet restores bioenergetic balance and extends lifespan in the presence of motor dysfunction and lumbar spinal cord motor neuron loss in TDP-43A315T mutant C57BL6/J mice. Dis Model Mech.

[37] Crane FL (2001). Biochemical functions of coenzyme Q10. J Am Coll Nutr.

[38] Crugnola V, Lamperti C, Lucchini V, Ronchi D, Peverelli L, Prelle A, Sciacco M, Bordoni A, Fassone E, Fortunato F, Corti S, Silani V, Bresolin N, Di Mauro S, Comi GP, Moggio M (2010). Mitochondrial respiratory chain dysfunction in muscle from patients with amyotrophic lateral sclerosis. Arch Neurol.

[39] Cudkowicz M, Bozik ME, Ingersoll EW, Miller R, Mitsumoto H, Shefner J, Moore DH, Schoenfeld D, Mather JL, Archibald D, Sullivan M, Amburgey C, Moritz J, Gribkoff VK (2011). The effects of dexpramipexole (KNS-760704) in individuals with amyotrophic lateral sclerosis. Nat Med.

[40] Cudkowicz ME, van den Berg LH, Shefner JM, Mitsumoto H, Mora JS, Ludolph A, Hardiman O, Bozik ME, Ingersoll EW, Archibald D, Meyers AL, Dong Y, Farwell WR, Kerr DA, Investigators E (2013). Dexpramipexole versus placebo for patients with amyotrophic lateral sclerosis (EMPOWER): a randomised, double-blind, phase 3 trial. Lancet Neurol.

[41] Dal Canto MC, Gurney ME (1994). Development of central nervous system pathology in a murine transgenic model of human amyotrophic lateral sclerosis. Am J Pathol.

[42] Dalakas MC, Hatazawa J, Brooks RA, Di Chiro G (1987). Lowered cerebral glucose utilization in amyotrophic lateral sclerosis. Ann Neurol.

[43] Daniel B, Green O, Viskind O, Gruzman A (2013). Riluzole increases the rate of glucose transport in L6 myotubes and NSC-34 motor neuron-like cells via AMPK pathway activation. Amyotroph Lateral Scler Frontotemporal Degener.

[44] Danzeisen R, Schwalenstoecker B, Gillardon F, Buerger E, Krzykalla V, Klinder K, Schild L, Hengerer B, Ludolph AC, Dorner-Ciossek C, Kussmaul L (2006). Targeted antioxidative and neuroprotective properties of the dopamine agonist pramipexole and its nondopaminergic enantiomer SND919CL2x [(+)2-amino-4,5,6,7-tetrahydro-6-Lpropylamino-benzathiazole dihydrochloride]. J Pharmacol Exp Ther.

[45] De Vos KJ, Chapman AL, Tennant ME, Manser C, Tudor EL, Lau KF, Brownlees J, Ackerley S, Shaw PJ, McLoughlin DM, Shaw CE, Leigh PN, Miller CCJ, Grierson AJ (2007). Familial amyotrophic lateral sclerosis-linked SOD1 mutants perturb fast axonal transport to reduce axonal mitochondria content. Hum Mol Genet.

[46] Derave W, Van Den Bosch L, Lemmens G, Eijnde BO, Robberecht W, Hespel P (2003). Skeletal muscle properties in a transgenic mouse model for amyotrophic lateral sclerosis: effects of creatine treatment. Neurobiol Dis.

[47] Diaz-Garcia CM, Mongeon R, Lahmann C, Koveal D, Zucker H, Yellen G (2017). Neuronal stimulation triggers neuronal glycolysis and not lactate uptake. Cell Metab.

[48] Dienel GA (2012). Brain lactate metabolism: the discoveries and the controversies. J Cereb Blood Flow Metab.

[49] Divakaruni AS, Wallace M, Buren C, Martyniuk K, Andreyev AY, Li E, Fields JA, Cordes T, Reynolds IJ, Bloodgood BL, Raymond LA, Metallo CM, Murphy AN (2017). Inhibition of the mitochondrial pyruvate carrier protects from excitotoxic neuronal death. J Cell Biol.

[50] Dodge JC, Treleaven CM, Fidler JA, Tamsett TJ, Bao C, Searles M, Taksir TV, Misra K, Sidman RL, Cheng SH, Shihabuddin LS (2013). Metabolic signatures of amyotrophic lateral sclerosis reveal insights into disease pathogenesis. Proc Natl Acad Sci USA.

[51] Dorst J, Cypionka J, Ludolph AC (2013). High-caloric food supplements in the treatment of amyotrophic lateral sclerosis: a prospective interventional study. Amyotroph Lateral Scler Frontotemporal Degener.

[52] Dorst J, Kuhnlein P, Hendrich C, Kassubek J, Sperfeld AD, Ludolph AC (2011). Patients with elevated triglyceride and cholesterol serum levels have a prolonged survival in amyotrophic lateral sclerosis. J Neurol.

[53] Doshi S, Gupta P, Kalb RG (2017). Genetic induction of hypometabolism by ablation of MC4R does not suppress ALS-like phenotypes in the G93A mutant SOD1 mouse model. Sci Rep.

[54] Dupuis L, Corcia P, Fergani A, De Gonzalez Aguilar JL, Bonnefont-Rousselot D, Bittar R, Seilhean D, Hauw JJ, Lacomblez L, Loeffler JP, Meininger V (2008). Dyslipidemia is a protective factor in amyotrophic lateral sclerosis. Neurology.

[55] Dupuis L, De Gonzalez Aguilar JL, Oudart H, de Tapia M, Barbeito L, Loeffler JP (2004). Mitochondria in amyotrophic lateral sclerosis: a trigger and a target. Neurodegener Dis.

[56] Dupuis L, Oudart H, Rene F, De Gonzalez Aguilar JL, Loeffler JP (2004). Evidence for defective energy homeostasis in amyotrophic lateral sclerosis: benefit of a high-energy diet in a transgenic mouse model. Proc Natl Acad Sci USA.

[57] Dupuis L, Pradat PF, Ludolph AC, Loeffler JP (2011). Energy metabolism in amyotrophic lateral sclerosis. Lancet Neurol.

[58] Echaniz-Laguna A, Zoll J, Ponsot E, N’Guessan B, Tranchant C, Loeffler JP, Lampert E (2006). Muscular mitochondrial function in amyotrophic lateral sclerosis is progressively altered as the disease develops: a temporal study in man. Exp Neurol.

[59] Esposito E, Capasso M, di Tomasso N, Corona C, Pellegrini F, Uncini A, Vitaglione P, Fogliano V, Piantelli M, Sensi SL (2007). Antioxidant strategies based on tomato-enriched food or pyruvate do not affect disease onset and survival in an animal model of amyotrophic lateral sclerosis. Brain Res.

[60] Feinberg AP, Fallin MD (2015). Epigenetics at the crossroads of genes and the environment. JAMA.

[61] Fendt SM, Verstreken P (2017). Neurons eat glutamate to stay alive. J Cell Biol.

[62] Fergani A, Oudart H, De Gonzalez Aguilar JL, Fricker B, Rene F, Hocquette JF, Meininger V, Dupuis L, Loeffler JP (2007). Increased peripheral lipid clearance in an animal model of amyotrophic lateral sclerosis. J Lipid Res.

[63] Ferrante RJ, Browne SE, Shinobu LA, Bowling AC, Baik MJ, MacGarvey U, Kowall NW, Brown RH, Beal MF (1997). Evidence of increased oxidative damage in both sporadic and familial amyotrophic lateral sclerosis. J Neurochem.

[64] Funfschilling U, Supplie LM, Mahad D, Boretius S, Saab AS, Edgar J, Brinkmann BG, Kassmann CM, Tzvetanova ID, Mobius W, Diaz F, Meijer D, Suter U, Hamprecht B, Sereda MW, Moraes CT, Frahm J, Goebbels S, Nave KA (2012). Glycolytic oligodendrocytes maintain myelin and long-term axonal integrity. Nature.

[65] Gallo V, Wark PA, Jenab M, Pearce N, Brayne C, Vermeulen R, Andersen PM, Hallmans G, Kyrozis A, Vanacore N, Vahdaninia M, Grote V, Kaaks R, Mattiello A, Bueno-de-Mesquita HB, Peeters PH, Travis RC, Petersson J, Hansson O, Arriola L, Jimenez-Martin JM, Tjonneland A, Halkjaer J, Agnoli C, Sacerdote C, Bonet C, Trichopoulou A, Gavrila D, Overvad K, Weiderpass E, Palli D, Quiros JR, Tumino R, Khaw KT, Wareham N, Barricante-Gurrea A, Fedirko V, Ferrari P, Clavel-Chapelon F, Boutron-Ruault MC, Boeing H, Vigl M, Middleton L, Riboli E, Vineis P (2013). Prediagnostic body fat and risk of death from amyotrophic lateral sclerosis: the EPIC cohort. Neurology.

[66] Girouard H, Iadecola C (2006). Neurovascular coupling in the normal brain and in hypertension, stroke, and Alzheimer disease. J Appl Physiol.

[67] Gorges M, Vercruysse P, Muller HP, Huppertz HJ, Rosenbohm A, Nagel G, Weydt P, Petersen A, Ludolph AC, Kassubek J, Dupuis L (2017). Hypothalamic atrophy is related to body mass index and age at onset in amyotrophic lateral sclerosis. J Neurol Neurosurg Psychiatry.

[68] Gribkoff VK, Bozik ME (2008). KNS-760704 [(6*R*)-4,5,6,7-tetrahydro-N6-propyl-2,6-benzothiazole-diamine dihydrochloride monohydrate] for the treatment of amyotrophic lateral sclerosis. CNS Neurosci Ther.

[69] Groeneveld GJ, Veldink JH, van der Tweel I, Kalmijn S, Beijer C, de Visser M, Wokke JH, Franssen H, van den Berg LH (2003). A randomized sequential trial of creatine in amyotrophic lateral sclerosis. Ann Neurol.

[70] Guo W, Naujock M, Fumagalli L, Vandoorne T, Baatsen P, Boon R, Ordovas L, Patel A, Welters M, Vanwelden T, Geens N, Tricot T, Benoy V, Steyaert J, Lefebvre-Omar C, Boesmans W, Jarpe M, Sterneckert J, Wegner F, Petri S, Bohl D, Vanden Berghe P, Robberecht W, Van Damme P, Verfaillie C, Van Den Bosch L (2017). HDAC6 inhibition reverses axonal transport defects in motor neurons derived from FUS-ALS patients. Nat Commun.

[71] Guzman M, Blazquez C (2004). Ketone body synthesis in the brain: possible neuroprotective effects. Prostaglandins Leukot Essent Fat Acids.

[72] Haidet-Phillips AM, Hester ME, Miranda CJ, Meyer K, Braun L, Frakes A, Song S, Likhite S, Murtha MJ, Foust KD, Rao M, Eagle A, Kammesheidt A, Christensen A, Mendell JR, Burghes AH, Kaspar BK (2011). Astrocytes from familial and sporadic ALS patients are toxic to motor neurons. Nat Biotechnol.

[73] Halim ND, McFate T, Mohyeldin A, Okagaki P, Korotchkina LG, Patel MS, Jeoung NH, Harris RA, Schell MJ, Verma A (2010). Phosphorylation status of pyruvate dehydrogenase distinguishes metabolic phenotypes of cultured rat brain astrocytes and neurons. Glia.

[74] Hardie DG, Schaffer BE, Brunet A (2016). AMPK: an energy-sensing pathway with multiple inputs and outputs. Trends Cell Biol.

[75] Harris JJ, Jolivet R, Attwell D (2012). Synaptic energy use and supply. Neuron.

[76] Hasel P, Dando O, Jiwaji Z, Baxter P, Todd AC, Heron S, Markus NM, McQueen J, Hampton DW, Torvell M, Tiwari SS, McKay S, Eraso-Pichot A, Zorzano A, Masgrau R, Galea E, Chandran S, Wyllie DJA, Simpson TI, Hardingham GE (2017). Neurons and neuronal activity control gene expression in astrocytes to regulate their development and metabolism. Nat Commun.

[77] Hatazawa J, Brooks RA, Dalakas MC, Mansi L, Di Chiro G (1988). Cortical motor-sensory hypometabolism in amyotrophic lateral sclerosis: a PET study. J Comput Assist Tomogr.

[78] Herrero-Mendez A, Almeida A, Fernandez E, Maestre C, Moncada S, Bolanos JP (2009). The bioenergetic and antioxidant status of neurons is controlled by continuous degradation of a key glycolytic enzyme by APC/C-Cdh1. Nat Cell Biol.

[79] Hua W, Young EC, Fleming ML, Gelles J (1997). Coupling of kinesin steps to ATP hydrolysis. Nature.

[80] Huang C, Tong J, Bi F, Wu Q, Huang B, Zhou H, Xia XG (2012). Entorhinal cortical neurons are the primary targets of FUS mislocalization and ubiquitin aggregation in FUS transgenic rats. Hum Mol Genet.

[81] Huisman MHB, Seelen M, van Doormaal PTC, de Jong SW, de Vries JHM, van der Kooi AJ, de Visser M, Schelhaas HJ, van den Berg LH, Veldink JH (2015). Effect of presymptomatic body mass index and consumption of fat and alcohol on amyotrophic lateral sclerosis. JAMA Neurol.

[82] Hyder F, Rothman DL, Bennett MR (2013). Cortical energy demands of signaling and nonsignaling components in brain are conserved across mammalian species and activity levels. Proc Natl Acad Sci USA.

[83] Isopi E, Granzotto A, Corona C, Bomba M, Ciavardelli D, Curcio M, Canzoniero LM, Navarra R, Lattanzio R, Piantelli M, Sensi SL (2015). Pyruvate prevents the development of age-dependent cognitive deficits in a mouse model of Alzheimer’s disease without reducing amyloid and tau pathology. Neurobiol Dis.

[84] Jaarsma D, Haasdijk ED, Grashorn JA, Hawkins R, van Duijn W, Verspaget HW, London J, Holstege JC (2000). Human Cu/Zn superoxide dismutase (SOD1) overexpression in mice causes mitochondrial vacuolization, axonal degeneration, and premature motoneuron death and accelerates motoneuron disease in mice expressing a familial amyotrophic lateral sclerosis mutant SOD1. Neurobiol Dis.

[85] Jakoby P, Schmidt E, Ruminot I, Gutierrez R, Barros LF, Deitmer JW (2014). Higher transport and metabolism of glucose in astrocytes compared with neurons: a multiphoton study of hippocampal and cerebellar tissue slices. Cereb Cortex.

[86] Jawaid A, Murthy SB, Wilson AM, Qureshi SU, Amro MJ, Wheaton M, Simpson E, Harati Y, Strutt AM, York MK, Schulz PE (2010). A decrease in body mass index is associated with faster progression of motor symptoms and shorter survival in ALS. Amyotroph Lateral Scler.

[87] Jawaid A, Salamone AR, Strutt AM, Murthy SB, Wheaton M, McDowell EJ, Simpson E, Appel SH, York MK, Schulz PE (2010). ALS disease onset may occur later in patients with pre-morbid diabetes mellitus. Eur J Neurol.

[88] Johnson BS, Snead D, Lee JJ, McCaffery JM, Shorter J, Gitler AD (2009). TDP-43 is intrinsically aggregation-prone, and amyotrophic lateral sclerosis-linked mutations accelerate aggregation and increase toxicity. J Biol Chem.

[89] Johnston CA, Stanton BR, Turner MR, Gray R, Blunt AH, Butt D, Ampong MA, Shaw CE, Leigh PN, Al-Chalabi A (2006). Amyotrophic lateral sclerosis in an urban setting: a population based study of inner city London. J Neurol.

[90] Jung C, Higgins CM, Xu Z (2002). Mitochondrial electron transport chain complex dysfunction in a transgenic mouse model for amyotrophic lateral sclerosis. J Neurochem.

[91] Kaneb HM, Sharp PS, Rahmani-Kondori N, Wells DJ (2011). Metformin treatment has no beneficial effect in a dose-response survival study in the SOD1(G93A) mouse model of ALS and is harmful in female mice. PLoS One.

[92] Kang SH, Li Y, Fukaya M, Lorenzini I, Cleveland DW, Ostrow LW, Rothstein JD, Bergles DE (2013). Degeneration and impaired regeneration of gray matter oligodendrocytes in amyotrophic lateral sclerosis. Nat Neurosci.

[93] Kashiwaya Y, Bergman C, Lee JH, Wan R, King MT, Mughal MR, Okun E, Clarke K, Mattson MP, Veech RL (2013). A ketone ester diet exhibits anxiolytic and cognition-sparing properties, and lessens amyloid and tau pathologies in a mouse model of Alzheimer’s disease. Neurobiol Aging.

[94] Katada S, Imhof A, Sassone-Corsi P (2012). Connecting threads: epigenetics and metabolism. Cell.

[95] Kaufmann P, Thompson JL, Levy G, Buchsbaum R, Shefner J, Krivickas LS, Katz J, Rollins Y, Barohn RJ, Jackson CE, Tiryaki E, Lomen-Hoerth C, Armon C, Tandan R, Rudnicki SA, Rezania K, Sufit R, Pestronk A, Novella SP, Heiman-Patterson T, Kasarskis EJ, Pioro EP, Montes J, Arbing R, Vecchio D, Barsdorf A, Mitsumoto H, Levin B, Group QS (2009). Phase II trial of CoQ10 for ALS finds insufficient evidence to justify phase III. Ann Neurol.

[96] Kelso GF, Porteous CM, Coulter CV, Hughes G, Porteous WK, Ledgerwood EC, Smith RA, Murphy MP (2001). Selective targeting of a redox-active ubiquinone to mitochondria within cells: antioxidant and antiapoptotic properties. J Biol Chem.

[97] Kesl SL, Poff AM, Ward NP, Fiorelli TN, Ari C, Van Putten AJ, Sherwood JW, Arnold P, D’Agostino DP (2016). Effects of exogenous ketone supplementation on blood ketone, glucose, triglyceride, and lipoprotein levels in Sprague–Dawley rats. Nutr Metab (Lond).

[98] Kim SM, Kim H, Kim JE, Park KS, Sung JJ, Kim SH, Lee KW (2011). Amyotrophic lateral sclerosis is associated with hypolipidemia at the presymptomatic stage in mice. PLoS One.

[99] Kioumourtzoglou MA, Rotem RS, Seals RM, Gredal O, Hansen J, Weisskopf MG (2015). Diabetes mellitus, obesity, and diagnosis of amyotrophic lateral sclerosis: a population-based study. JAMA Neurol.

[100] Kira Y, Nishikawa M, Ochi A, Sato E, Inoue M (2006). l-Carnitine suppresses the onset of neuromuscular degeneration and increases the life span of mice with familial amyotrophic lateral sclerosis. Brain Res.

[101] Klivenyi P, Ferrante RJ, Matthews RT, Bogdanov MB, Klein AM, Andreassen OA, Mueller G, Wermer M, Kaddurah-Daouk R, Beal MF (1999). Neuroprotective effects of creatine in a transgenic animal model of amyotrophic lateral sclerosis. Nat Med.

[102] Kong JM, Xu ZS (1998). Massive mitochondrial degeneration in motor neurons triggers the onset of amyotrophic lateral sclerosis in mice expressing a mutant SOD1. J Neurosci.

[103] Lacomblez L, Doppler V, Beucler I, Costes G, Salachas F, Raisonnier A, Le Forestier N, Pradat PF, Bruckert E, Meininger V (2002). APOE: a potential marker of disease progression in ALS. Neurology.

[104] LaManna JC, Salem N, Puchowicz M, Erokwu B, Koppaka S, Flask C, Lee Z (2009). Ketones suppress brain glucose consumption. Adv Exp Med Biol.

[105] Langhans W, Berthoud HR, Westerterp-Plantenga M (2016). Introduction to ‘All roads take to the brain: neural control of energy homeostasis in health and disease’. Int J Obes.

[106] Le Masson G, Przedborski S, Abbott LF (2014). A computational model of motor neuron degeneration. Neuron.

[107] Le WD, Jankovic J, Xie W, Appel SH (2000). Antioxidant property of pramipexole independent of dopamine receptor activation in neuroprotection. J Neural Transm (Vienna).

[108] Lederer CW, Torrisi A, Pantelidou M, Santama N, Cavallaro S (2007). Pathways and genes differentially expressed in the motor cortex of patients with sporadic amyotrophic lateral sclerosis. BMC Genom.

[109] Lee Y, Morrison BM, Li Y, Lengacher S, Farah MH, Hoffman PN, Liu Y, Tsingalia A, Jin L, Zhang PW, Pellerin L, Magistretti PJ, Rothstein JD (2012). Oligodendroglia metabolically support axons and contribute to neurodegeneration. Nature.

[110] Lim MA, Selak MA, Xiang Z, Krainc D, Neve RL, Kraemer BC, Watts JL, Kalb RG (2012). Reduced activity of AMP-activated protein kinase protects against genetic models of motor neuron disease. J Neurosci.

[111] Lindauer E, Dupuis L, Muller HP, Neumann H, Ludolph AC, Kassubek J (2013). Adipose tissue distribution predicts survival in amyotrophic lateral sclerosis. PLoS One.

[112] Liu L, MacKenzie KR, Putluri N, Maletic-Savatic M, Bellen HJ (2017). The glia-neuron lactate shuttle and elevated ROS promote lipid synthesis in neurons and lipid droplet accumulation in glia via APOE/D. Cell Metab.

[113] Liu L, Zhang K, Sandoval H, Yamamoto S, Jaiswal M, Sanz E, Li Z, Hui J, Graham BH, Quintana A, Bellen HJ (2015). Glial lipid droplets and ROS induced by mitochondrial defects promote neurodegeneration. Cell.

[114] Liu YJ, Ju TC, Chen HM, Jang YS, Lee LM, Lai HL, Tai HC, Fang JM, Lin YL, Tu PH, Chern Y (2015). Activation of AMP-activated protein kinase alpha1 mediates mislocalization of TDP-43 in amyotrophic lateral sclerosis. Hum Mol Genet.

[115] Llorente-Folch I, Rueda CB, Amigo I, del Arco A, Saheki T, Pardo B, Satrustegui J (2013). Calcium-regulation of mitochondrial respiration maintains ATP homeostasis and requires ARALAR/AGC1-malate aspartate shuttle in intact cortical neurons. J Neurosci.

[116] Locasale JW, Cantley LC (2011). Metabolic flux and the regulation of mammalian cell growth. Cell Metab.

[117] Loeffler JP, Picchiarelli G, Dupuis L, De Gonzalez Aguilar JL (2016). The role of skeletal muscle in amyotrophic lateral sclerosis. Brain Pathol.

[118] Lopez-Gonzalez R, Lu Y, Gendron TF, Karydas A, Tran H, Yang D, Petrucelli L, Miller BL, Almeida S, Gao FB (2016). Poly(GR) in C9ORF72-related ALS/FTD compromises mitochondrial function and increases oxidative stress and DNA damage in iPSC-derived motor neurons. Neuron.

[119] Lu C, Thompson CB (2012). Metabolic regulation of epigenetics. Cell Metab.

[120] Machler P, Wyss MT, Elsayed M, Stobart J, Gutierrez R, von Faber-Castell A, Kaelin V, Zuend M, San Martin A, Romero-Gomez I, Baeza-Lehnert F, Lengacher S, Schneider BL, Aebischer P, Magistretti PJ, Barros LF, Weber B (2016). In vivo evidence for a lactate gradient from astrocytes to neurons. Cell Metab.

[121] Magistretti PJ, Allaman I (2015). A cellular perspective on brain energy metabolism and functional imaging. Neuron.

[122] Magrane J, Cortez C, Gan WB, Manfredi G (2014). Abnormal mitochondrial transport and morphology are common pathological denominators in SOD1 and TDP43 ALS mouse models. Hum Mol Genet.

[123] Mak TW, Grusdat M, Duncan GS, Dostert C, Nonnenmacher Y, Cox M, Binsfeld C, Hao Z, Brustle A, Itsumi M, Jager C, Chen Y, Pinkenburg O, Camara B, Ollert M, Bindslev-Jensen C, Vasiliou V, Gorrini C, Lang PA, Lohoff M, Harris IS, Hiller K, Brenner D (2017). Glutathione primes T cell metabolism for inflammation. Immunity.

[124] Mamczur P, Borsuk B, Paszko J, Sas Z, Mozrzymas J, Wisniewski JR, Gizak A, Rakus D (2015). Astrocyte-neuron crosstalk regulates the expression and subcellular localization of carbohydrate metabolism enzymes. Glia.

[125] Manconi M, Casetta I, Govoni V, Cesnik E, Ferini-Strambi L, Granieri E (2003). Pramipexole in restless legs syndrome: evaluation by suggested immobilization test. J Neurol.

[126] Marin B, Desport JC, Kajeu P, Jesus P, Nicolaud B, Nicol M, Preux PM, Couratier P (2011). Alteration of nutritional status at diagnosis is a prognostic factor for survival of amyotrophic lateral sclerosis patients. J Neurol Neurosurg Psychiatry.

[127] Mariosa D, Kamel F, Bellocco R, Ye W, Fang F (2015). Association between diabetes and amyotrophic lateral sclerosis in Sweden. Eur J Neurol.

[128] Matthews RT, Yang L, Browne S, Baik M, Beal MF (1998). Coenzyme Q10 administration increases brain mitochondrial concentrations and exerts neuroprotective effects. Proc Natl Acad Sci USA.

[129] Mattiazzi M, D’Aurelio M, Gajewski CD, Martushova K, Kiaei M, Beal MF, Manfredi G (2002). Mutated human SOD1 causes dysfunction of oxidative phosphorylation in mitochondria of transgenic mice. J Biol Chem.

[130] Menzies FM, Ince PG, Shaw PJ (2002). Mitochondrial involvement in amyotrophic lateral sclerosis. Neurochem Int.

[131] Miquel E, Cassina A, Martinez-Palma L, Bolatto C, Trias E, Gandelman M, Radi R, Barbeito L, Cassina P (2012). Modulation of astrocytic mitochondrial function by dichloroacetate improves survival and motor performance in inherited amyotrophic lateral sclerosis. PLoS One.

[132] Miquel E, Cassina A, Martinez-Palma L, Souza JM, Bolatto C, Rodriguez-Bottero S, Logan A, Smith RA, Murphy MP, Barbeito L, Radi R, Cassina P (2014). Neuroprotective effects of the mitochondria-targeted antioxidant MitoQ in a model of inherited amyotrophic lateral sclerosis. Free Radic Biol Med.

[133] Miyazaki K, Masamoto K, Morimoto N, Kurata T, Mimoto T, Obata T, Kanno I, Abe K (2012). Early and progressive impairment of spinal blood flow-glucose metabolism coupling in motor neuron degeneration of ALS model mice. J Cereb Blood Flow Metab.

[134] Moller A, Bauer CS, Cohen RN, Webster CP, De Vos KJ (2017). Amyotrophic lateral sclerosis-associated mutant SOD1 inhibits anterograde axonal transport of mitochondria by reducing Miro1 levels. Hum Mol Genet.

[135] Nagel G, Peter RS, Rosenbohm A, Koenig W, Dupuis L, Rothenbacher D, Ludolph AC (2017). Adipokines, C-reactive protein and amyotrophic lateral sclerosis—results from a population-based ALS registry in Germany. Sci Rep.

[136] Neumann M, Sampathu DM, Kwong LK, Truax AC, Micsenyi MC, Chou TT, Bruce J, Schuck T, Grossman M, Clark CM, McCluskey LF, Miller BL, Masliah E, Mackenzie IR, Feldman H, Feiden W, Kretzschmar HA, Trojanowski JQ, Lee VM (2006). Ubiquitinated TDP-43 in frontotemporal lobar degeneration and amyotrophic lateral sclerosis. Science.

[137] Nijssen J, Comley LH, Hedlund E (2017). Motor neuron vulnerability and resistance in amyotrophic lateral sclerosis. Acta Neuropathol.

[138] O’Reilly EJ, Wang H, Weisskopf MG, Fitzgerald KC, Falcone G, McCullough ML, Thun M, Park Y, Kolonel LN, Ascherio A (2013). Premorbid body mass index and risk of amyotrophic lateral sclerosis. Amyotroph Lateral Scler Frontotemporal Degener.

[139] Onofrj M, Ciccocioppo F, Varanese S, di Muzio A, Calvani M, Chiechio S, Osio M, Thomas A (2013). Acetyl-l-carnitine: from a biological curiosity to a drug for the peripheral nervous system and beyond. Exp Rev Neurother.

[140] Pagani M, Chio A, Valentini MC, Oberg J, Nobili F, Calvo A, Moglia C, Bertuzzo D, Morbelli S, De Carli F, Fania P, Cistaro A (2014). Functional pattern of brain FDG-PET in amyotrophic lateral sclerosis. Neurology.

[141] Palamiuc L, Schlagowski A, Ngo ST, Vernay A, Dirrig-Grosch S, Henriques A, Boutillier AL, Zoll J, Echaniz-Laguna A, Loeffler JP, Rene F (2015). A metabolic switch toward lipid use in glycolytic muscle is an early pathologic event in a mouse model of amyotrophic lateral sclerosis. EMBO Mol Med.

[142] Park JH, Hong YH, Kim HJ, Kim SM, Kim MJ, Park KS, Sung JJ, Lee KW (2007). Pyruvate slows disease progression in a G93A SOD1 mutant transgenic mouse model. Neurosci Lett.

[143] Patel A, Malinovska L, Saha S, Wang J, Alberti S, Krishnan Y, Hyman AA (2017). ATP as a biological hydrotrope. Science.

[144] Patel AB, Lai JC, Chowdhury GM, Hyder F, Rothman DL, Shulman RG, Behar KL (2014). Direct evidence for activity-dependent glucose phosphorylation in neurons with implications for the astrocyte-to-neuron lactate shuttle. Proc Natl Acad Sci USA.

[145] Patel BP, Safdar A, Raha S, Tarnopolsky MA, Hamadeh MJ (2010). Caloric restriction shortens lifespan through an increase in lipid peroxidation, inflammation and apoptosis in the G93A mouse, an animal model of ALS. PLoS One.

[146] Pedersen WA, Mattson MP (1999). No benefit of dietary restriction on disease onset or progression in amyotrophic lateral sclerosis Cu/Zn-superoxide dismutase mutant mice. Brain Res.

[147] Pellerin L, Magistretti PJ (1994). Glutamate uptake into astrocytes stimulates aerobic glycolysis: a mechanism coupling neuronal activity to glucose utilization. Proc Natl Acad Sci USA.

[148] Pellerin L, Magistretti PJ (2004). Neuroenergetics: calling upon astrocytes to satisfy hungry neurons. Neuroscientist.

[149] Peter RS, Rosenbohm A, Dupuis L, Brehme T, Kassubek J, Rothenbacher D, Nagel G, Ludolph AC (2017). Life course body mass index and risk and prognosis of amyotrophic lateral sclerosis: results from the ALS registry Swabia. Eur J Epidemiol.

[150] Philips T, Bento-Abreu A, Nonneman A, Haeck W, Staats K, Geelen V, Hersmus N, Kusters B, Van Den Bosch L, Van Damme P, Richardson WD, Robberecht W (2013). Oligodendrocyte dysfunction in the pathogenesis of amyotrophic lateral sclerosis. Brain.

[151] Philips T, Rothstein JD (2017). Oligodendroglia: metabolic supporters of neurons. J Clin Invest.

[152] Pierre K, Pellerin L (2005). Monocarboxylate transporters in the central nervous system: distribution, regulation and function. J Neurochem.

[153] Popova I, Malkov A, Ivanov AI, Samokhina E, Buldakova S, Gubkina O, Osypov A, Muhammadiev RS, Zilberter T, Molchanov M, Paskevich S, Zilberter M, Zilberter Y (2017). Metabolic correction by pyruvate halts acquired epilepsy in multiple rodent models. Neurobiol Dis.

[154] Pradat PF, Bruneteau G, Gordon PH, Dupuis L, Bonnefont-Rousselot D, Simon D, Salachas F, Corcia P, Frochot V, Lacorte JM, Jardel C, Coussieu C, Le Forestier N, Lacomblez L, Loeffler JP, Meininger V (2010). Impaired glucose tolerance in patients with amyotrophic lateral sclerosis. Amyotroph Lateral Scler.

[155] Prior R, Van Helleputte L, Benoy V, Van Den Bosch L (2017). Defective axonal transport: a common pathological mechanism in inherited and acquired peripheral neuropathies. Neurobiol Dis.

[156] Quaegebeur A, Segura I, Schmieder R, Verdegem D, Decimo I, Bifari F, Dresselaers T, Eelen G, Ghosh D, Davidson SM, Schoors S, Broekaert D, Cruys B, Govaerts K, De Legher C, Bouche A, Schoonjans L, Ramer MS, Hung G, Bossaert G, Cleveland DW, Himmelreich U, Voets T, Lemmens R, Bennett CF, Robberecht W, De Bock K, Dewerchin M, Ghesquiere B, Fendt SM, Carmeliet P (2016). Deletion or inhibition of the oxygen sensor PHD1 protects against ischemic stroke via reprogramming of neuronal metabolism. Cell Metab.

[157] Raman R, Allen SP, Goodall EF, Kramer S, Ponger LL, Heath PR, Milo M, Hollinger HC, Walsh T, Highley JR, Olpin S, McDermott CJ, Shaw PJ, Kirby J (2015). Gene expression signatures in motor neurone disease fibroblasts reveal dysregulation of metabolism, hypoxia-response and RNA processing functions. Neuropathol Appl Neurobiol.

[158] Rodriguez-Rodriguez P, Fernandez E, Almeida A, Bolanos JP (2012). Excitotoxic stimulus stabilizes PFKFB3 causing pentose-phosphate pathway to glycolysis switch and neurodegeneration. Cell Death Differ.

[159] Rosen DR, Siddique T, Patterson D, Figlewicz DA, Sapp P, Hentati A, Donaldson D, Goto J, O’Regan JP, Deng HX (1993). Mutations in Cu/Zn superoxide dismutase gene are associated with familial amyotrophic lateral sclerosis. Nature.

[160] Rosenfeld J, King RM, Jackson CE, Bedlack RS, Barohn RJ, Dick A, Phillips LH, Chapin J, Gelinas DF, Lou JS (2008). Creatine monohydrate in ALS: effects on strength, fatigue, respiratory status and ALSFRS. Amyotroph Lateral Scler.

[161] Rothstein JD, Van Kammen M, Levey AI, Martin LJ, Kuncl RW (1995). Selective loss of glial glutamate transporter GLT-1 in amyotrophic lateral sclerosis. Ann Neurol.

[162] Ruminot I, Schmalzle J, Leyton B, Barros LF, Deitmer JW (2017). Tight coupling of astrocyte energy metabolism to synaptic activity revealed by genetically encoded FRET nanosensors in hippocampal tissue. J Cereb Blood Flow Metab.

[163] Sasaki S, Horie Y, Iwata M (2007). Mitochondrial alterations in dorsal root ganglion cells in sporadic amyotrophic lateral sclerosis. Acta Neuropathol.

[164] Sasaki S, Iwata M (1996). Ultrastructural study of synapses in the anterior horn neurons of patients with amyotrophic lateral sclerosis. Neurosci Lett.

[165] Sawada H (2017). Clinical efficacy of edaravone for the treatment of amyotrophic lateral sclerosis. Exp Opin Pharmacother.

[166] Scafidi S, Racz J, Hazelton J, McKenna MC, Fiskum G (2010). Neuroprotection by acetyl-l-carnitine after traumatic injury to the immature rat brain. Dev Neurosci.

[167] Schnitzer MJ, Block SM (1997). Kinesin hydrolyses one ATP per 8-nm step. Nature.

[168] Schonfeld P, Reiser G (2013). Why does brain metabolism not favor burning of fatty acids to provide energy? Reflections on disadvantages of the use of free fatty acids as fuel for brain. J Cereb Blood Flow Metab.

[169] Schousboe A, Scafidi S, Bak LK, Waagepetersen HS, McKenna MC (2014). Glutamate metabolism in the brain focusing on astrocytes. Adv Neurobiol.

[170] Shan X, Chiang PM, Price DL, Wong PC (2010). Altered distributions of Gemini of coiled bodies and mitochondria in motor neurons of TDP-43 transgenic mice. Proc Natl Acad Sci USA.

[171] Shannon KM, Bennett JP, Friedman JH, The Pramipexole Study Group (1997). Efficacy of pramipexole, a novel dopamine agonist, as monotherapy in mild to moderate Parkinson’s disease. Neurology.

[172] Shaw PJ, Eggett CJ (2000). Molecular factors underlying selective vulnerability of motor neurons to neurodegeneration in amyotrophic lateral sclerosis. J Neurol.

[173] Shefner JM, Cudkowicz ME, Schoenfeld D, Conrad T, Taft J, Chilton M, Urbinelli L, Qureshi M, Zhang H, Pestronk A, Caress J, Donofrio P, Sorenson E, Bradley W, Lomen-Hoerth C, Pioro E, Rezania K, Ross M, Pascuzzi R, Heiman-Patterson T, Tandan R, Mitsumoto H, Rothstein J, Smith-Palmer T, MacDonald D, Burke D, Consortium N (2004). A clinical trial of creatine in ALS. Neurology.

[174] Sheng ZH (2017). The interplay of axonal energy homeostasis and mitochondrial trafficking and anchoring. Trends Cell Biol.

[175] Shenk JC, Liu J, Fischbach K, Xu K, Puchowicz M, Obrenovich ME, Gasimov E, Alvarez LM, Ames BN, Lamanna JC, Aliev G (2009). The effect of acetyl-l-carnitine and *R*-alpha-lipoic acid treatment in ApoE4 mouse as a model of human Alzheimer’s disease. J Neurol Sci.

[176] Sherman MS, Pillai A, Jackson A, Heiman-Patterson T (2004). Standard equations are not accurate in assessing resting energy expenditure in patients with amyotrophic lateral sclerosis. JPEN J Parenter Enter Nutr.

[177] Shimazu T, Hirschey MD, Newman J, He W, Shirakawa K, Le Moan N, Grueter CA, Lim H, Saunders LR, Stevens RD, Newgard CB, Farese RV, de Cabo R, Ulrich S, Akassoglou K, Verdin E (2013). Suppression of oxidative stress by beta-hydroxybutyrate, an endogenous histone deacetylase inhibitor. Science.

[178] Shults CW, Beal MF, Song D, Fontaine D (2004). Pilot trial of high dosages of coenzyme Q10 in patients with Parkinson’s disease. Exp Neurol.

[179] Shults CW, Oakes D, Kieburtz K, Beal MF, Haas R, Plumb S, Juncos JL, Nutt J, Shoulson I, Carter J, Kompoliti K, Perlmutter JS, Reich S, Stern M, Watts RL, Kurlan R, Molho E, Harrison M, Lew M, Parkinson Study G (2002). Effects of coenzyme Q10 in early Parkinson disease: evidence of slowing of the functional decline. Arch Neurol.

[180] Siklos L, Engelhardt J, Harati Y, Smith RG, Joo F, Appel SH (1996). Ultrastructural evidence for altered calcium in motor nerve terminals in amyotropic lateral sclerosis. Ann Neurol.

[181] Sills MA, Forsythe WI, Haidukewych D, MacDonald A, Robinson M (1986). The medium chain triglyceride diet and intractable epilepsy. Arch Dis Child.

[182] Smith EF, Shaw PJ, De Vos KJ (2017). The role of mitochondria in amyotrophic lateral sclerosis. Neurosci Lett.

[183] So E, Mitchell JC, Memmi C, Chennell G, Vizcay-Barrena G, Allison L, Shaw CE, Vance C (2017). Mitochondrial abnormalities and disruption of the neuromuscular junction precede the clinical phenotype and motor neuron loss in hFUSWT transgenic mice. Hum Mol Genet.

[184] Sohn JW, Elmquist JK, Williams KW (2013). Neuronal circuits that regulate feeding behavior and metabolism. Trends Neurosci.

[185] Sotelo-Silveira JR, Lepanto P, Elizondo V, Horjales S, Palacios F, Martinez-Palma L, Marin M, Beckman JS, Barbeito L (2009). Axonal mitochondrial clusters containing mutant SOD1 in transgenic models of ALS. Antioxid Redox Signal.

[186] Stevens PR, Gawryluk JW, Hui L, Chen X, Geiger JD (2014). Creatine protects against mitochondrial dysfunction associated with HIV-1 Tat-induced neuronal injury. Curr HIV Res.

[187] Swinnen B, Robberecht W (2014). The phenotypic variability of amyotrophic lateral sclerosis. Nat Rev Neurol.

[188] Szelechowski M, Amoedo N, Obre E, Leger C, Allard L, Bonneu M, Claverol S, Lacombe D, Oliet S, Chevallier S, Le Masson G, Rossignol R (2018). Metabolic reprogramming in amyotrophic lateral sclerosis. Sci Rep.

[189] Tauskela JS (2007). MitoQ–a mitochondria-targeted antioxidant. IDrugs.

[190] Taylor JP, Brown RH, Cleveland DW (2016). Decoding ALS: from genes to mechanism. Nature.

[191] Tefera TW, Wong Y, Barkl-Luke ME, Ngo ST, Thomas NK, McDonald TS, Borges K (2016). Triheptanoin protects motor neurons and delays the onset of motor symptoms in a mouse model of amyotrophic lateral sclerosis. PLoS One.

[192] Thevenet J, De Marchi U, Domingo JS, Christinat N, Bultot L, Lefebvre G, Sakamoto K, Descombes P, Masoodi M, Wiederkehr A (2016). Medium-chain fatty acids inhibit mitochondrial metabolism in astrocytes promoting astrocyte-neuron lactate and ketone body shuttle systems. FASEB J.

[193] Tress O, Maglione M, May D, Pivneva T, Richter N, Seyfarth J, Binder S, Zlomuzica A, Seifert G, Theis M, Dere E, Kettenmann H, Willecke K (2012). Panglial gap junctional communication is essential for maintenance of myelin in the CNS. J Neurosci.

[194] Turner MR, Goldacre R, Ramagopalan S, Talbot K, Goldacre MJ (2013). Autoimmune disease preceding amyotrophic lateral sclerosis: an epidemiologic study. Neurology.

[195] Valbuena GN, Rizzardini M, Cimini S, Siskos AP, Bendotti C, Cantoni L, Keun HC (2016). Metabolomic analysis reveals increased aerobic glycolysis and amino acid deficit in a cellular model of Amyotrophic lateral sclerosis. Mol Neurobiol.

[196] Van Hall G, Stromstad M, Rasmussen P, Jans O, Zaar M, Gam C, Quistorff B, Secher NH, Nielsen HB (2009). Blood lactate is an important energy source for the human brain. J Cereb Blood Flow Metab.

[197] Van Laere K, Vanhee A, Verschueren J, De Coster L, Driesen A, Dupont P, Robberecht W, Van Damme P (2014). Value of 18fluorodeoxyglucose–positron-emission tomography in amyotrophic lateral sclerosis: a prospective study. JAMA Neurol.

[198] Vandoorne T, De Smet S, Ramaekers M, Van Thienen R, De Bock K, Clarke K, Hespel P (2017). Intake of a ketone ester drink during recovery from exercise promotes mTORC1 signaling but not glycogen resynthesis in human muscle. Front Physiol.

[199] Veech RL (2004). The therapeutic implications of ketone bodies: the effects of ketone bodies in pathological conditions: ketosis, ketogenic diet, redox states, insulin resistance, and mitochondrial metabolism. Prostaglandins Leukot Essent Fat Acids.

[200] Vendelin M, Eimre M, Seppet E, Peet N, Andrienko T, Lemba M, Engelbrecht J, Seppet EK, Saks VA (2004). Intracellular diffusion of adenosine phosphates is locally restricted in cardiac muscle. Mol Cell Biochem.

[201] Vercruysse P, Sinniger J, El Oussini H, Scekic-Zahirovic J, Dieterle S, Dengler R, Meyer T, Zierz S, Kassubek J, Fischer W, Dreyhaupt J, Grehl T, Hermann A, Grosskreutz J, Witting A, Van Den Bosch L, Spreux-Varoquaux O, Ludolph AC, Dupuis L, Group GAS (2016). Alterations in the hypothalamic melanocortin pathway in amyotrophic lateral sclerosis. Brain.

[202] Vieira FG, LaDow E, Moreno A, Kidd JD, Levine B, Thompson K, Gill A, Finkbeiner S, Perrin S (2014). Dexpramipexole is ineffective in two models of ALS related neurodegeneration. PLoS One.

[203] Vielhaber S, Winkler K, Kirches E, Kunz D, Buchner M, Feistner H, Elger CE, Ludolph AC, Riepe MW, Kunz WS (1999). Visualization of defective mitochondrial function in skeletal muscle fibers of patients with sporadic amyotrophic lateral sclerosis. J Neurol Sci.

[204] Volkenhoff A, Weiler A, Letzel M, Stehling M, Klambt C, Schirmeier S (2015). Glial glycolysis is essential for neuronal survival in drosophila. Cell Metab.

[205] Wang W, Li L, Lin WL, Dickson DW, Petrucelli L, Zhang T, Wang X (2013). The ALS disease-associated mutant TDP-43 impairs mitochondrial dynamics and function in motor neurons. Hum Mol Genet.

[206] Wang XS, Simmons Z, Liu W, Boyer PJ, Connor JR (2006). Differential expression of genes in amyotrophic lateral sclerosis revealed by profiling the post mortem cortex. Amyotroph Lateral Scler.

[207] Wiedemann FR, Manfredi G, Mawrin C, Beal MF, Schon EA (2002). Mitochondrial DNA and respiratory chain function in spinal cords of ALS patients. J Neurochem.

[208] Wills AM, Hubbard J, Macklin EA, Glass J, Tandan R, Simpson EP, Brooks B, Gelinas D, Mitsumoto H, Mozaffar T, Hanes GP, Ladha SS, Heiman-Patterson T, Katz J, Lou JS, Mahoney K, Grasso D, Lawson R, Yu H, Cudkowicz M, Network MDACR (2014). Hypercaloric enteral nutrition in patients with amyotrophic lateral sclerosis: a randomised, double-blind, placebo-controlled phase 2 trial. Lancet.

[209] Wong M, Martin LJ (2010). Skeletal muscle-restricted expression of human SOD1 causes motor neuron degeneration in transgenic mice. Hum Mol Genet.

[210] Wong PC, Pardo CA, Borchelt DR, Lee MK, Copeland NG, Jenkins NA, Sisodia SS, Cleveland DW, Price DL (1995). An adverse property of a familial ALS-linked SOD1 mutation causes motor neuron disease characterized by vacuolar degeneration of mitochondria. Neuron.

[211] Writing G, Edaravone ALSSG (2017). Safety and efficacy of edaravone in well defined patients with amyotrophic lateral sclerosis: a randomised, double-blind, placebo-controlled trial. Lancet Neurol.

[212] Wyss MT, Jolivet R, Buck A, Magistretti PJ, Weber B (2011). In vivo evidence for lactate as a neuronal energy source. J Neurosci.

[213] Xu YF, Gendron TF, Zhang YJ, Lin WL, D’Alton S, Sheng H, Casey MC, Tong J, Knight J, Yu X, Rademakers R, Boylan K, Hutton M, McGowan E, Dickson DW, Lewis J, Petrucelli L (2010). Wild-type human TDP-43 expression causes TDP-43 phosphorylation, mitochondrial aggregation, motor deficits, and early mortality in transgenic mice. J Neurosci.

[214] Yamanaka K, Chun SJ, Boillee S, Fujimori-Tonou N, Yamashita H, Gutmann DH, Takahashi R, Misawa H, Cleveland DW (2008). Astrocytes as determinants of disease progression in inherited amyotrophic lateral sclerosis. Nat Neurosci.

[215] Yang JW, Kim SM, Kim HJ, Kim JE, Park KS, Kim SH, Lee KW, Sung JJ (2013). Hypolipidemia in patients with amyotrophic lateral sclerosis: a possible gender difference?. J Clin Neurol.

[216] Yang YM, Gupta SK, Kim KJ, Powers BE, Cerqueira A, Wainger BJ, Ngo HD, Rosowski KA, Schein PA, Ackeifi CA, Arvanites AC, Davidow LS, Woolf CJ, Rubin LL (2013). A small molecule screen in stem-cell-derived motor neurons identifies a kinase inhibitor as a candidate therapeutic for ALS. Cell Stem Cell.

[217] Zala D, Hinckelmann MV, Yu H, Lyra da Cunha MM, Liot G, Cordelieres FP, Marco S, Saudou F (2013). Vesicular glycolysis provides on-board energy for fast axonal transport. Cell.

[218] Zhang F, Wang W, Siedlak SL, Liu Y, Liu J, Jiang K, Perry G, Zhu X, Wang X (2015). Miro1 deficiency in amyotrophic lateral sclerosis. Front Aging Neurosci.

[219] Zhang Y, Chen K, Sloan SA, Bennett ML, Scholze AR, O’Keeffe S, Phatnani HP, Guarnieri P, Caneda C, Ruderisch N, Deng S, Liddelow SA, Zhang C, Daneman R, Maniatis T, Barres BA, Wu JQ (2014). An RNA-sequencing transcriptome and splicing database of glia, neurons, and vascular cells of the cerebral cortex. J Neurosci.

[220] Zhao W, Varghese M, Vempati P, Dzhun A, Cheng A, Wang J, Lange D, Bilski A, Faravelli I, Pasinetti GM (2012). Caprylic triglyceride as a novel therapeutic approach to effectively improve the performance and attenuate the symptoms due to the motor neuron loss in ALS disease. PLoS One.

[221] Zhao W, Varghese M, Yemul S, Pan Y, Cheng A, Marano P, Hassan S, Vempati P, Chen F, Qian X, Pasinetti GM (2011). Peroxisome proliferator activator receptor gamma coactivator-1alpha (PGC-1alpha) improves motor performance and survival in a mouse model of amyotrophic lateral sclerosis. Mol Neurodegener.

[222] Zhao Z, Lange DJ, Voustianiouk A, MacGrogan D, Ho L, Suh J, Humala N, Thiyagarajan M, Wang J, Pasinetti GM (2006). A ketogenic diet as a potential novel therapeutic intervention in amyotrophic lateral sclerosis. BMC Neurosci.

[223] Zilberter Y, Gubkina O, Ivanov AI (2015). A unique array of neuroprotective effects of pyruvate in neuropathology. Front Neurosci.

[224] Zinman L, Sadeghi R, Gawel M, Patton D, Kiss A (2008). Are statin medications safe in patients with ALS?. Amyotroph Lateral Scler.

